# Bright light therapy versus physical exercise to prevent co-morbid depression and obesity in adolescents and young adults with attention-deficit / hyperactivity disorder: study protocol for a randomized controlled trial

**DOI:** 10.1186/s13063-017-2426-1

**Published:** 2018-02-26

**Authors:** Jutta S. Mayer, Katharina Hees, Juliane Medda, Oliver Grimm, Philip Asherson, Mariano Bellina, Michael Colla, Pol Ibáñez, Elena Koch, Antonio Martinez-Nicolas, Adrià Muntaner-Mas, Anna Rommel, Nanda Rommelse, Saskia de Ruiter, Ulrich W. Ebner-Priemer, Meinhard Kieser, Francisco B. Ortega, Johannes Thome, Jan K. Buitelaar, Jonna Kuntsi, J. Antoni Ramos-Quiroga, Andreas Reif, Christine M. Freitag

**Affiliations:** 1 Department of Child and Adolescent Psychiatry, Psychosomatics and Psychotherapy, University Hospital Frankfurt, Goethe University, Deutschordenstr. 50, 60528 Frankfurt am Main, Germany; 2 Institute of Medical Biometry and Informatics, University Hospital Heidelberg, Heidelberg, Germany; 3 Department of Psychiatry, Psychosomatic Medicine and Psychotherapy, University Hospital Frankfurt, Goethe University, Frankfurt am Main, Germany; 4 King’s College London, Social, Genetic and Developmental Psychiatry Centre, Institute of Psychiatry, Psychology and Neuroscience, London, UK; 5 Department of Psychiatry, Hospital Universitari Vall d’Hebron, Barcelona, Catalonia Spain; 6 Psychiatric Genetics Unit, Group of Psychiatry, Mental Health and Addiction, Vall d’Hebron Research Institute (VHIR), Barcelona, Catalonia Spain; 7 Department of Psychiatry, School of Medicine, University of Rostock, Rostock, Germany; 8 Mental mHealth Lab, Department of Sport and Sport Science, Karlsruhe Institute of Technology (KIT), Karlsruhe, Germany; 9 PROFITH “PROmoting FITness and Health through physical activity” research group, Department of Physical Education and Sports, Faculty of Sport Sciences, University of Granada, Granada, Spain; 10 Chronobiology Research Group, Department of Physiology, Faculty of Biology, University of Murcia. Campus Mare Nostrum. IUIE. IMIB-Arrixaca. Ciber Fragilidad y Envejecimiento Saludable (CIBERFES), Madrid, Spain; 11 Physical Activity and Exercise Sciences Research Group, University of Balearic Islands, Palma, Spain; 12 Department of Psychiatry, Radboudumc, Nijmegen, The Netherlands; 13 Karakter Child and Adolescent Psychiatry University Centre, Nijmegen, The Netherlands; 14 Department of Cognitive Neuroscience, Donders Institute for Brain, Cognition and Behaviour, Radboudumc, Nijmegen, The Netherlands; 15 Biomedical Network Research Centre on Mental Health (CIBERSAM), Instituto de Salud Carlos III, Madrid, Spain; 16 Department of Psychiatry and Forensic Medicine, Universitat Autònoma de Barcelona, Barcelona, Catalonia Spain

**Keywords:** Attention-deficit / hyperactivity disorder, Co-morbidity, Depression, Obesity, Bright light therapy, Exercise

## Abstract

**Background:**

The risk for major depression and obesity is increased in adolescents and adults with attention-deficit / hyperactivity disorder (ADHD) and adolescent ADHD predicts adult depression and obesity. Non-pharmacological interventions to treat and prevent these co-morbidities are urgently needed. Bright light therapy (BLT) improves day–night rhythm and is an emerging therapy for major depression. Exercise intervention (EI) reduces obesity and improves depressive symptoms. To date, no randomized controlled trial (RCT) has been performed to establish feasibility and efficacy of these interventions targeting the prevention of co-morbid depression and obesity in ADHD. We hypothesize that the two manualized interventions in combination with mobile health-based monitoring and reinforcement will result in less depressive symptoms and obesity compared to treatment as usual in adolescents and young adults with ADHD.

**Methods:**

This trial is a prospective, pilot phase-IIa, parallel-group RCT with three arms (two add-on treatment groups [BLT, EI] and one treatment as usual [TAU] control group). The primary outcome variable is change in the Inventory of Depressive Symptomatology total score (observer-blinded assessment) between baseline and ten weeks of intervention. This variable is analyzed with a mixed model for repeated measures approach investigating the treatment effect with respect to all three groups. A total of 330 participants with ADHD, aged 14 – < 30 years, will be screened at the four study centers. To establish effect sizes, the sample size was planned at the liberal significance level of α = 0.10 (two-sided) and the power of 1-β = 80% in order to find medium effects. Secondary outcomes measures including change in obesity, ADHD symptoms, general psychopathology, health-related quality of life, neurocognitive function, chronotype, and physical fitness are explored after the end of the intervention and at the 12-week follow-up.

**Discussion:**

This is the first pilot RCT on the use of BLT and EI in combination with mobile health-based monitoring and reinforcement targeting the prevention of co-morbid depression and obesity in adolescents and young adults with ADHD. If at least medium effects can be established with regard to the prevention of depressive symptoms and obesity, a larger scale confirmatory phase-III trial may be warranted.

**Trial registration:**

German Clinical Trials Register, DRKS00011666. Registered on 9 February 2017. ClinicalTrials.gov, NCT03371810. Registered on 13 December 2017.

**Electronic supplementary material:**

The online version of this article (10.1186/s13063-017-2426-1) contains supplementary material, which is available to authorized users.

## Background

Attention-deficit / hyperactivity disorder (ADHD) is a neurodevelopmental disorder characterized by age-inappropriate hyperactivity, impulsivity, and inattention with onset in early childhood and a high rate of persistence into adulthood [1]. With a ~ 5% prevalence in childhood and ~ 3% in adulthood, ADHD is among the most common psychiatric disorders [2, 3]. Being a prevalent neurodevelopmental disorder with childhood onset, ADHD is also often the entry point into a trajectory defined by a high risk for co-morbid conditions. Co-morbidity is a hallmark of adult ADHD [4]: around 85% of adults with ADHD suffer from at least one co-morbid psychiatric disorder including, most often, mood (~ 60%), anxiety (~ 30%), substance use disorders (~ 45%), and personality disorders (~ 35%) [5]. Children and adolescents with ADHD are at high risk of developing these psychiatric conditions, specifically depression, when they reach adulthood [6, 7]. Furthermore, the prevalence of obesity, which is about 40% higher in children and adolescents with ADHD compared to individuals without ADHD, is further increased during adulthood (about 70% higher in adults with ADHD compared with individuals without ADHD) [8–10]. Thus, obesity—accompanied by an increased risk of metabolic syndrome and cardiovascular disease—can be regarded as an important somatic sequel of ADHD [11]. Co-morbid psychiatric and somatic conditions significantly increase disease burden leading to higher rates of detrimental outcomes on health (i.e. more than doubling mortality rates) and socioeconomic status [12]. Therefore, effective treatments of ADHD’s co-morbid disorders, specifically depression and obesity, are urgently needed. Moreover, prevention of these co-morbid conditions during the potentially sensitive phase of adolescence and young adulthood [6, 13] is of upmost importance.

Stimulant medication (e.g. methylphenidate) is the first-line pharmacologic treatment of the core symptoms of ADHD [14]. However, their effects on co-morbid depression and obesity are largely unclear due to a lack of randomized controlled studies (RCTs). Also, the effects of non-stimulant medication (e.g. atomoxetine) on these co-morbid symptoms have been rarely studied [15].

With regard to depression, evidence derived from animal models suggests that early exposure to stimulants may increase the long-term risk of depressive-like behaviors [16, 17]. In contrast, retrospective and prospective clinical studies following adolescents with ADHD found none or protective effects of stimulants on the risk of later depression [18–22]. Stimulant medication has also been associated with reduced rates of concurrent depression and suicide-related events in patients with ADHD [22, 23]. In contrast, atomoxetine has not been shown to be effective in improving co-occurring depressive symptoms in adolescents with ADHD [15, 24].

With regard to obesity, a meta-analysis of cross-sectional studies found that rates of obesity were decreased by about 40% in pharmacologically treated patients (mainly with stimulants) compared with non-pharmacologically treated ADHD patients [8]. However, although an anorexigenic effect of stimulants has been reported [25, 26], to our knowledge, RCTs and prospective clinical studies targeting (1) the effects of pharmacological ADHD treatment on disordered eating and obesity in adolescents and adults with ADHD and (2) the risk for developing obesity in adulthood are lacking.

In conclusion, the available evidence for potential protective effects of pharmacological ADHD treatments on co-occurring depressive symptoms and obesity is largely limited in adolescents and young adults with ADHD. In addition, non-adherence to medication typically increases during adolescence [27–29], further complicating effective treatment and prevention of ADHD co-morbidities during this particularly risky developmental phase. Therefore, alternative or adjunct non-pharmacological interventions to treat and prevent ADHD and its co-occurring symptoms are needed for this population.

Only few studies have evaluated the effectiveness of psychosocial interventions in adolescents with ADHD taking co-morbid conditions into account. The available evidence suggests some benefit of skills training coupled with parent and teacher training for academic and organizational skills, whereas effects on core ADHD as well as on co-occurring emotional and behavioral symptoms have been inconsistent [30]. Manualized cognitive behavioral therapy (CBT) combining operant (i.e. skills training) with cognitive strategies (i.e. cognitive restructuring) is another treatment option that has been assessed in RCTs in adolescents and adults with ADHD. The findings of the few studies consistently reported beneficial effects on core ADHD symptoms [31–34] which were accompanied by additional improvements of co-morbid symptoms, such as depressive, anxiety, and oppositional-defiant symptoms, organizational skills, and functional impairment [31, 32, 35]. Preliminary data suggest that adolescents with co-morbid depression and anxiety might benefit most from manualized CBT [36], a finding that warrants further investigation.

Taken together, the development of effective pharmacological and non-pharmacological treatments to improve and prevent co-morbid depression and obesity in adolescents and adults with ADHD is still in its infancy. Guidelines for treatment and prevention of co-morbid depression and obesity in adolescents and young adults with ADHD are not yet available. Therefore, a wider range of treatment and prevention approaches need to be evaluated in RCTs and these interventions should directly target the known pathophysiological mechanisms of ADHD and its co-morbidities. Following these goals, the present phase-IIa trial aimed at establishing feasibility and effect sizes of two kinds of non-pharmacological interventions—physical exercise (exercise intervention [EI]) and bright light therapy (BLT)—in order to prevent the development and progression of depression and obesity in adolescents and young adults with ADHD.

Physical exercise is thought to directly modulate a dopamine (DA) dysregulation [37] which has been established as a key pathophysiological mechanism underlying ADHD but also plays a role in mood disorders (especially in anhedonic behavior) [38] and obesity, conceptualized as addictive food intake [39]. Following the idea of a shared DA dysregulation that can be modulated by physical exercise, therapeutic effects of this intervention on ADHD symptoms and co-morbid depression and obesity can be hypothesized.

Previously, the effectiveness of physical exercise in reducing depressive symptoms has been demonstrated in mildly and moderately depressed adults [40, 41] and adolescents [42]. It is also known that physical exercise and higher cardiorespiratory fitness attenuates the health risks of obesity [43, 44]. Physical exercise interventions have been successfully implemented in programs to prevent obesity in children [45, 46] and have been shown to effectively reduce weight in overweight and obese adults and adolescents [47, 48]. Moreover, physical fitness has been associated with improved cognitive function [49]. With regard to ADHD, some evidence suggests that physical exercise improves neurocognitive function in children with ADHD [50], and therefore has been discussed as a potential protective factor for ADHD [51]. Specifically, physical exercise may release DA in the brain, improving attention and cognition [52, 53], and therefore it may be used to regulate hyperactivity as well as inattentive symptoms in people with ADHD [54, 55]. These previous findings strongly suggest that physical exercise bears the potential of improving and/or preventing the core symptoms of ADHD; however, its effects on obesity and depression await systematic investigation in patients with ADHD [56].

BLT is thought to modulate the circadian (CIRCA) system dysfunctions [57, 58]—another key pathophysiological mechanism possibly linking ADHD to co-morbid symptoms of depression and obesity [59–62]. Following the idea of a shared CIRCA dysregulation possibly associated with the striatal dopaminergic system [63] that can be modulated by BLT, therapeutic effects of this intervention on ADHD symptoms as well as co-morbid depression and obesity can be expected.

In ADHD, circadian system dysfunctions are indicated by phase delays in the sleep/wake cycle with changes in diurnal preference towards greater eveningness, nocturnal rise in melatonin, and early morning increase in cortisol [57, 58, 64, 65]. Physiologically, when administered in the early morning, BLT suppresses the night-time melatonin production [66] and decreases the cortisol levels that usually peak after waking up [57]. Hence, with morning light administration, the wake-time can be shifted to an earlier time (phase advance) and circadian rhythms can be stabilized [67]. BLT has been shown to be efficacious for the treatment of seasonal and non-seasonal depression in adults and adolescents [68–71], whereas findings on the prevention of seasonal affective disorder have been inconclusive [72]. Accumulating evidence also suggests its efficacy in eating disorders and obesity [73]. In patients with ADHD, a recent study demonstrated that morning BLT advanced sleep timing which was associated with decreased ADHD symptoms, specifically hyperactivity and impulsivity [74]. One open-label trial also examined the potential of BLT on improving co-morbid depressive symptoms [75]. Three weeks of morning BLT advanced the circadian phase in ADHD adults as measured by a questionnaire and led to significant reductions in both subjective and objective measures of core ADHD symptoms as well as depressive symptoms. Together, these findings suggest that chronobiological therapies bear substantial innovation potential, but RCTs are needed to systematically test their feasibility and effectiveness on improving ADHD and co-morbid depression and obesity in adolescents and young adults.

Importantly, targeting adolescents and young adults with these interventions implies specific problems, as this age group usually has little motivation for lifestyle change. Therefore, the PROUD trial will make use of cutting edge mobile technology, which is generally viewed very favorably by this age group, assuming that this might booster motivation. Both interventions will be supported by a mobile health (m-health) application that monitors physical exercise, light exposure, and related parameters and feeds them back to the user to improve motivation for change. A recent meta-analysis has concluded that reinforcement-based exercise interventions using m-health approaches improve effects on weight loss in obesity [76]. Although commercial apps for mental disorders which have no empirical evidence are omnipresent, scientific studies using m-health applications as a tool to monitor and reinforce interventions in mental disorders, specifically ADHD, are largely lacking [54, 77].

In conclusion, the aim of the present multicenter, prospective, pilot, observer-blinded, parallel-group (allocation ratio 1:1:1), phase-IIa RCT is to establish feasibility and effect sizes of two add-on ten-week interventions—EI and BLT in combination with m-Health-based reinforcement—targeting the prevention of the development and progression of co-morbid depression and obesity in adolescents and young adults aged 14 – < 30 years with ADHD. Both interventions are risk-free, cost-effective, easy-to-use, and portable, and therefore can be easily implemented in the daily life of adolescents and young adults. If at least medium effects can be established with regard to the prevention of depressive symptoms and obesity, a larger scale confirmatory phase-III trial may be warranted.

It is hypothesized that the two manualized ten-week interventions will result in a smaller increase of depressive symptoms and obesity compared to a treatment as usual (TAU) control condition. In addition, pre-existing depressive symptoms and obesity are expected to decrease after ten weeks of either BLT or EI compared to TAU. TAU includes ten weeks of stable pharmacotherapy, group-based or individual CBT (not including elements of BLT or EI). To assess the stability of these secondary prevention effects, follow-up assessment will be done 12 weeks after the end of the intervention. Furthermore, immediate and long-term intervention effects on core ADHD symptoms, general psychopathological symptoms, health-related quality of life, neurocognitive function, chronotype, body-related measures such as blood pressure and heart rate, physical fitness, and hormone concentrations will be assessed. Variables possibly moderating treatment effect, including age, sex, medication, physical fitness and activity, daily light exposure, chronotype, and mood regulation will be explored as well as variables possibly mediating treatment effects, such as reward processing, stress reactivity, and compliance with the intervention.

## Methods

This protocol is presented in accordance with the 2013 SPIRIT (Standard Protocol Items: Recommendations for Interventional Trials) Statement (See Additional file 1 for the populated SPIRIT Checklist) [78].

### Study setting

The trial is performed by close cooperation of four large European clinical centers: Goethe University Hospital Frankfurt, Germany (Department of Child and Adolescent Psychiatry, Psychosomatics and Psychotherapy, and Department of Psychiatry, Psychosomatic Medicine and Psychotherapy); Radboud University Medical Centre, Nijmegen, The Netherlands (Karakter Child and Adolescent Psychiatry, and Department of Psychiatry); Vall d’Hebron Research Institute (Group of Psychiatry, Mental Health and Addiction), Barcelona, Catalonia, Spain; and Institute of Psychiatry, Psychology and Neuroscience, King’s College London (Social, Genetic and Developmental Psychiatry Centre), UK.

### Eligibility criteria

Participants’ inclusion and exclusion criteria are listed in Table 1. All participants must be aged 14 – < 30 years, meet DSM-5 criteria for a lifetime history of childhood onset ADHD as well as current ADHD criteria established by a specialist in the field, and show an intelligence quotient (IQ) ≥ 75. TAU will be allowed in all groups. TAU includes stable psychopharmacotherapy for ADHD (stimulant and non-stimulant medication), stable medication for chronic medical conditions not interfering with interventions, individual or group-based psychotherapy or family support. With regard to co-morbid psychiatric disorders, participants with any severe psychiatric disorder (especially bipolar disorder, schizophrenia, autism spectrum disorder, schizoaffective disorder, organic psychiatric disorder [current or lifetime], borderline personality and substance use disorder or dependency) other than the co-morbid conditions explicitly studied, or patients requiring additional psychopharmacotherapy or psychiatric intervention, including day-care/inpatient treatment at start of the study, are excluded. With regard to co-morbid medical and neurological conditions, it is essential that participants have no severe condition interfering with or not allowing either BLT (e.g. diagnosed eye condition or other diseases with effects on the retina such as Diabetes mellitus, or recent eye surgery) or EI (e.g. heart disease, high blood pressure, injuries). Also, participants are not allowed to use antipsychotic, antiepileptic, or photo-sensitizing medication. Only a single participation in the trial is allowed.

**Table 1:** Inclusion and exclusion criteria of the PROUD trial

Inclusion criteria	All participants must meet DSM-5 criteria for a lifetime history of childhood onset ADHD (DSM-5 314.00, 314.01) as well as current ADHD criteria established by a specialist in the field
	Age 14 – < 30 years
	Written informed consent of the legal caretakers of the participant (age < 18 years) and, if possible, written assent of the participant (age < 18 years) himself
	Written informed consent of the of the participant (age ≥ 18 years)
	Stable TAU comprising pharmacotherapy, group-based or individual CBT (not including elements of BLT or EI)
	Normal or corrected to normal vision
	Ability to understand, read, write, and speak fluently in the language of the study site
	Ability to regularly and reliably attend appointments
Exclusion criteria	IQ < 75 (measured by WAIS-IV or WISC-IV vocabulary and matrix reasoning subtests)
	Any severe co-morbid psychiatric disorder other than the co-morbid conditions explicitly studied with necessary additional psychopharmacotherapy or psychiatric intervention involving day-care/inpatient treatment at start of study, especially a diagnosis of bipolar disorder, schizophrenia, autism spectrum disorder, schizoaffective disorder or organic psychiatric disorder (current or lifetime)
	Any severe medical or neurological condition interfering with interventions
	Any severe medical or neurological condition not allowing BLT or EI
	Use of antipsychotic or anti-epileptic medication, photo-sensitizing medication (e.g. lithium, St. John’s Wort)
	Substance use disorder (DSM-5) or dependency (DSM-5)
	History of epilepsy
	Acute suicidal ideation
	Pregnancy
	Participant is related to the investigator or study staff
	Participation in other clinical trials and observation period of competing trials (participation in other studies is permitted if the respective study is a no-medication or psychotherapy trial and if its aims do not interfere with the aims of the present study)
	No participant will be allowed to enroll in this trial more than once

*WAIS* Wechsler Adult Intelligence Scale, *WISC* Wechsler Intelligence Scale for Children, *IQ* intelligence quotient, *CBT* cognitive behavioral therapy

### Interventions

#### BLT

Light therapy consists of a daily (except Sunday) 30-min exposure of white light without ultraviolet (UV) components in the morning or evening for ten weeks in total provided by special 10,000 lx light boxes that supply broadband, UV-filtered light, specifically designed for BLT (Philips EnergyLight HF 3419). The exact time of day of implementation (either during the morning between 06:00 and 08:00 am or the evening between 06:00 and 08:00 pm and) is determined by the type of chronotype (morning or evening type) of each study participant determined by the Morningness–Eveningness Questionnaire (MEQ) [79]. The light therapy device is handed over by trained psychologists or psychiatrists along with an introduction to the operation and how to carry out the light therapy at home. When receiving light therapy, participants sit approximately 50–75 cm from the light box, facing the illumination and glancing at the light occasionally. Participants are encouraged to read, watch TV, or work on a computer while the bright light is directed at their eyes. Monitoring and feedback is realized with the m-health system comprising a smartphone (Motorola Moto G3) equipped with the BLT app (movisensXS software, movisens GmbH, 2016) and an activity sensor (LightMove 3 wrist, movisens GmbH, 2016, Fig. 1) equipped with a light sensor to monitor the light exposure of the participant. Participants wear the LightMove 3 wrist daily (24 h). Participants are introduced to the usage of the m-health app by trained psychologists or psychiatrists and a user’s guide is handed over. The m-health app allows monitoring when participants start and stop daily BLT; in addition, the BLT is monitored by the light sensor. The m-health app also sends an acoustic signal to remind participants of their BLT and provide them with individual feedback every day. Physicians or therapists will not receive this feedback. Therapists will assess participants’ compliance based on interviews at T3.

**Fig. 1:**
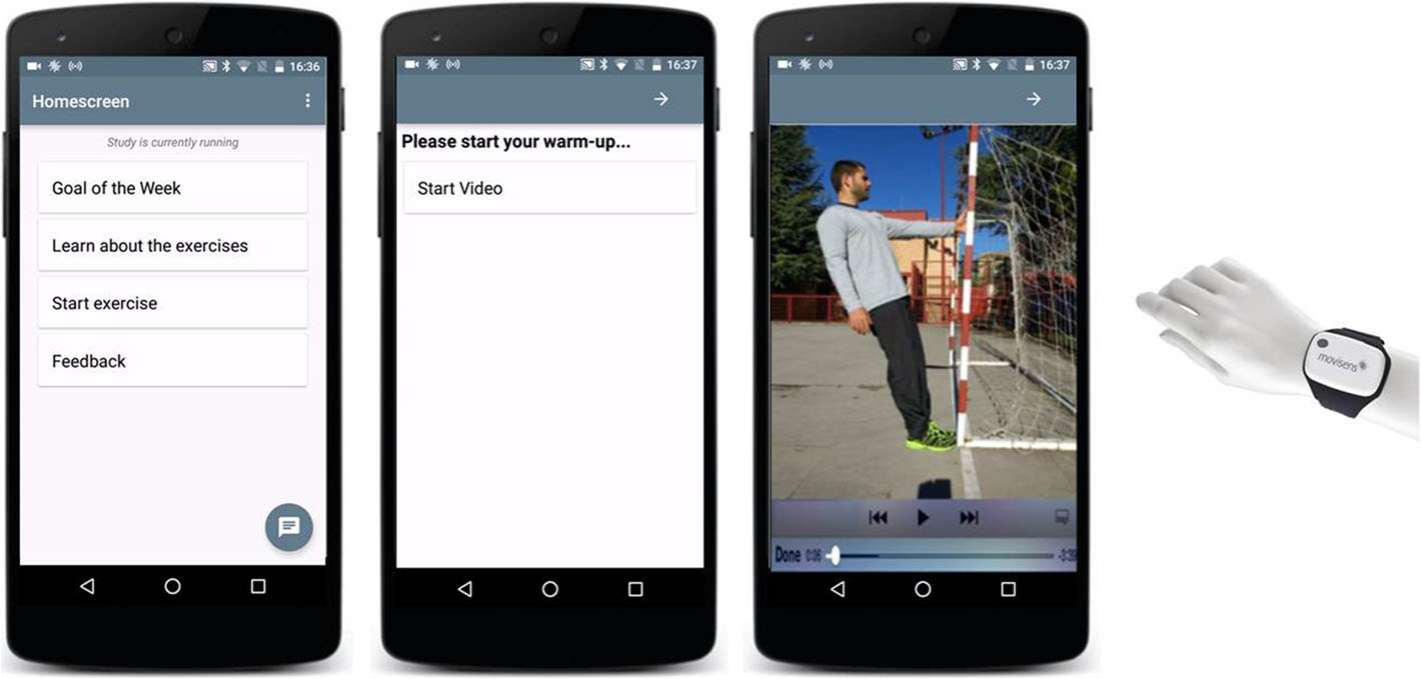
M-Health system consisting of the smartphone and the sensor (adapted with permission of movisens). Example from the EI intervention. The “home screen” of the movisensXS app shows four different buttons: (1) Goal of the week; (2) Learn about the exercises; (3) Start exercise; (4) Feedback. If participants press button three (Start exercise), the exercise videos are played

#### EI

In developing the exercise manual and learning videos, we followed the internationally accepted physical activity guidelines [80]. These guidelines are based on a systematic review of > 2000 references and are the basic platform for designing any exercise intervention. In accordance with the guidelines, most of the time during the exercise sessions is spent in aerobic exercise of moderate-to-vigorous intensity and strength activities. The physical exercise intervention consists in training three days a week for ten weeks. Participants will perform three days of proposed aerobic activities and on two of these days they will also do muscle-strengthening exercises. Specifically, a training day consists of: (1) a 5-min warm-up period; (2) 10–35 min of muscle-strength training on two of the three days; (3) 20–40 min of aerobic training; and (4) 5 min of flexibility/stretching cool-down. During the course of the ten weeks, the duration and intensity of the exercises increases gradually by combining number of exercises, repetitions, rests, and frequency.

(1) The warm-up consists of doing an activity at a slower speed or lower intensity. A warm-up before moderate or vigorous intensity aerobic activity allows a gradual increase in heart rate and breathing at the start of the episode of activity. Warm-up includes light walking and joint mobilization of the upper (neck, shoulders) and lower limbs (hips, knees, and ankles). (2) The muscle-strength training includes whole-body exercises, which incorporate push-ups, front plank, lunge, chair squat, hip thrust, etc. (3) Aerobic activities are physical activities in which people move their large muscles in a rhythmic manner for a sustained period. Aerobic activity makes a person’s heart beat more rapidly to meet the demands of the body’s movement. Running, brisk walking, bicycling, playing basketball, dancing, and swimming are all examples of aerobic activities. (4) A cool-down after activity allows a gradual decrease at the end of the episode. The cool-down period includes breathing, stretching, and relaxing exercises.

The participants can decide which days of the week they want to perform the physical exercises. Participants are recommended to complete training sessions separated by 48-h rest periods whenever possible. Not all participants will have the same fitness level at the beginning of the intervention. Consequently, and in order to ensure compliance by all participants, we prescribe three physical exercise programs of differing intensity based on the participant’s baseline cardiorespiratory fitness. In particular, we use the Chester Step Test, which is included in the pre-test measurements for that task, to assess the baseline cardiorespiratory fitness level. The Chester Step Test can predict maximal oxygen uptake (VO2max). Based on the value obtained in the Chester Step Test, participants are allocated to one of the three exercises programs (light, moderate, or high intensity). All three exercise programs have the same warm-up and cool-down exercises. The participants do the same exercise program proposed at baseline during the ten-week intervention.

Instruction, monitoring, and feedback are realized by the m-health system including a smartphone (Motorola Moto G3) equipped with the m-health app (movisensXS software, movisens GmbH, 2016), SD cards to store the exercise videos, as well as an activity sensor (LightMove 3 wrist, movisens GmbH, 2016) equipped with a mobile sensor for the acquisition of physical activity (LightMove 3 wrist, movisens GmbH, 2016, Fig. 1). The sensor is equipped with a Bluetooth Smart interface and offers the possibility of doing online analysis of data on the sensor. The sensor records the raw data of three-dimensional (3D) acceleration, barometric air pressure, and temperature. From these data, secondary parameters such as activity class, body position, steps, energy expenditure, and metabolic equivalents can be calculated with the movisens DataAnalyzer software. The sensor can be fixed with a band at the wrist. Participants are asked to wear the sensor daily (24 h) during the ten-week intervention period. Participants are introduced to the usage of the m-Health app by trained psychologists or psychiatrists and a user’s guide is handed over.

Strengthening exercises are presented in the form of video sessions on the smartphones which are executed while watching the videos. The videos present an exercise specialist who carries out the different physical exercises proposed and subtitles showing graphical description, intensity, and rest of each exercise. The m-health app also allows monitoring when participants start and stop their aerobic and strengthening exercises; in addition, the EI is monitored by the activity sensor. Acoustic signals to remind participants of their EI as well as individual feedback (a reward summary including information on duration of the excises, movement acceleration intensity, and number of steps accompanied by a motivational message) are provided by the m-health app at the end of each day. Physicians or therapists will not receive this feedback. Therapists will assess participants’ compliance based on interviews at T3.

#### Criteria for discontinuing allocated interventions for a given trial participant

Generally, both, BLT and EI, are considered as safe interventions with no specific, relevant risk conferred to the trial participants. The BLT device implemented in this study (Chronolux Medic-4) uses UV and infrared (IR) filtered therapeutic light (10,000 lx) and is thus safe for the eyes and skin. If side effects occur (e.g. nausea, headache, eyestrain), they are usually mild and short-lived [81]. In rare circumstances, BLT can trigger a manic episode in bipolar disorder, which is therefore an exclusion criterion [81]. Regarding the EI arm of the intervention, it should be said that the risks associated with exercise are directly related to the “dose” of exercise and top athletes are at a high risk of suffering different types of injuries. However, in this study, the exercise administered will be recreational and only small injuries (e.g. ankle sprain) might occur with a comparable probability to school recess (in the case of adolescent participants) or in any daily activity. If any undesirable effect occurs, this will be reported as an adverse event (AE) and reported to the local primary investigator and to the principal investigator (PI) who will decide about withdrawal of participants from the clinical trial (see Additional file 2). All ongoing AEs/serious adverse events (SAEs) of withdrawn participants will be followed up until no more signs and symptoms are verifiable or the participant is in a stable condition or the participant has taken back his/her approval for medical follow-up.

#### Strategies to improve adherence to intervention protocols and any procedures for monitoring adherence

To ensure comparability of the BLT and EI interventions between the four different centers, detailed manuals have been developed that: provide a standardized psycho-education element to explain the basic elements of the respective therapy and its mode of action; detail the frequency and duration of the respective therapy; detail the m-health-based electronic monitoring, reinforcement, and coaching part; and provide the clinical therapist with standardized motivational interviewing skills to improve participants’ compliance with the intervention. Participants’ compliance with the intervention (BLT and EI) is monitored with the m-health app and the light and activity sensor throughout the ten weeks of intervention. Therapists have no access to this information but will assess participants’ compliance based on interviews at T3.

#### Relevant concomitant care and interventions that are permitted or prohibited during the trial

Psychotropic medication is started or changed at least four weeks before randomization and needs to remain stable (mg/kg body weight) throughout the intervention and the three-month follow-up of the study (with the exception of dose adjustment to body weight changes). The following psychotropic medication is allowed as single or combined treatment: any ADHD-specific medication, antidepressive treatment, and low dose neuroleptic treatment to control aggressive behavior or mood swings. In addition, stable medication for the treatment of chronic conditions as allergies, asthma, enuresis, sleeping problems, and intermitting medication for acute infections or pain is allowed. Pharmacological treatment is documented at each time of assessment (T1–T5, see Fig. 3) and psychotropic medication effects on treatment outcome will be explored in analyzing study outcomes. Any individual-based (e.g. CBT that does not include elements of BLT and EI, school-based intervention, occupational, language, psychomotor therapy) as well as family-based intervention is allowed. Any additional treatment is documented exactly (kind of intervention, frequency, etc.). The following concomitant treatments are not permitted during the trial: additional EI and additional BLT. Relevant additional treatments administered to the participants on entry to the trial or at any time during the trial are regarded as concomitant treatments and are documented on the appropriate pages of the case report form (CRF).

### Outcome measures

The primary outcome measure is the change in the clinician-rated Inventory of Depressive Symptomatology (IDS-C_30_) [82] total score (observer-blinded assessment) between baseline (T2) and after the end of the intervention (T4, primary endpoint, see Fig. 3). The IDS-C_30_ rating includes all DSM-5 diagnostic criterion items for major depressive disorder (e.g. mood, vegetative, psychomotor, and cognitive symptoms) as well as commonly associated symptoms such as anxiety, irritability, melancholic, and atypical symptom features to assess the severity of depressive symptoms over the last seven days. Items are rated on a 4-point Likert scale based on the information obtained during a semi-structured interview. The total score range is 0–84. The psychometric properties of the IDS-C_30_ and its sensitivity to change with interventions have been well established in different study samples and RCTs [82–84]. Parallel versions exist in English, German, Spanish, and Dutch. The IDS is under investigation in adolescent patients; however, a standardization for individuals aged 14–17 years is still lacking. Because changes in raw scores are assessed in this study, the IDS-C_30_ is considered a valid measure in adolescents.

The secondary outcome measures aim to assess intervention effects on depressive symptoms at the 12-week follow-up (T5) and to differentially assess changes in obesity, health-related quality of life, ADHD symptoms, general psychopathology, chronotype, neurocognitive function, body-related measures, and physical fitness between baseline (T2), the end of intervention/ TAU (T4), and 12-week follow-up (T5). In addition, secondary outcome measures include several parameters measured with the m-health app between the one-week baseline and one-week post-intervention assessment. Also, hormone concentrations will be assessed in a Frankfurt subsample at T2 and T4. All scales and questionnaires as well as physical fitness tests and neurocognitive tests have been frequently used in clinical and non-clinical research and have been validated in adults and mostly also in adolescents (see Additional file 3). For all assessments, parallel versions exist in the languages of the four study sites. Secondary outcome measures are described in detail in Additional file 3.

### Participant timeline

**Fig. 2:**
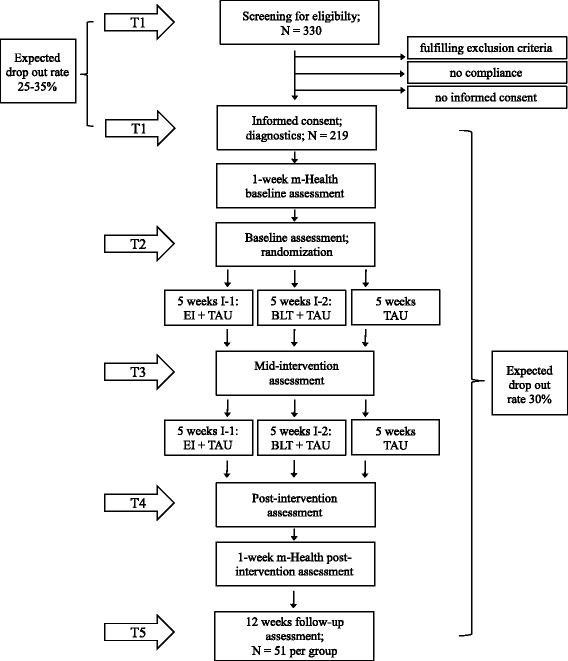
Trial time flow. T, time-point; I, intervention; EI, exercise intervention; BLT, bright light therapy; TAU, treatment as usual

**Fig. 3:**
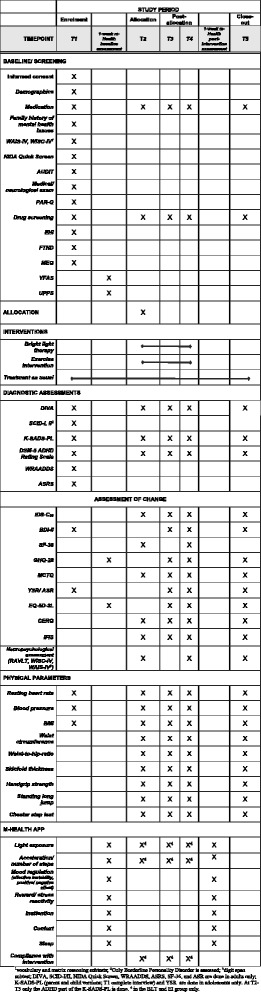
Schedule of enrolment, interventions, and assessments at the different time-points (T1–T5)

The trial time flow is shown in Figs. 2 and 3. At T1, the diagnosis of ADHD is established by performing structured clinical interviews. The Kiddie-Schedule for Affective Disorders and Schizophrenia - Present and Lifetime Version (K-SADS-PL) [85] is used to assess ADHD symptoms and co-morbid conditions in adolescents. To obtain as much information as possible for high valid diagnoses, both with regard to externalizing and internalizing conditions, the K-SADS-PL will be conducted separately with the adolescent and at least one primary caregiver. For the final rating, the trained clinician will take both sources of information into account. In adults, the Diagnostic Interview for ADHD in adults (DIVA) [86] is administered to assess ADHD symptoms and the Structured Clinical Interview for DSM-IV Axis I and II Disorders (SCID-I and II) [87] to assess co-morbid conditions. The Adult ADHD Self-Report Scale (ASRS) [88] and the Wender-Reimherr Adult ADHD Symptom Rating Scale (WRAADDS) [89] are used to substantiate diagnosis. All interviews and questionnaires will be adjusted to DSM-5 criteria. After having established the diagnosis of ADHD and having completed screening for eligibility based on interviews/questionnaires (Alcohol Use Identification Test [AUDIT] [90], National Institute on Drug Abuse [NIDA] Quick Screen [91], Physical Activity Readiness Questionnaire [PAR-Q] [92], physical examination, and IQ tests [Wechsler Adult Intelligence Scale, WAIS-IV [93]/ Wechsler Intelligence Scale for Children, WISC-IV [94]], informed consent is obtained. Trial-specific assessments are done at T1 after informed consent has been obtained and m-health will be introduced to participants and their parents (if applicable) (T1, duration = approximately 5 h for adults and 3.5 h for children/parents including breaks). Within two weeks after T1, the one-week baseline assessment with the m-Health system takes place at home. During this week, participants will wear the light and movement sensors on two working days and on Saturday and Sunday (always 24 h) and they will be asked to answer questionnaires (regarding mood regulation, reward and stress reactivity, sleep behavior, inattention, and context) 12 times a day (duration = 1 min each) provided by the m-health app. A subset of adult participants from Frankfurt are also asked to collect saliva on one day of the one-week period at home ten times over 24 h (immediately after awakening: approximately at 07:00, 30 min after wakening: approximately at 7:30 am, 11.00 am, 6.00 pm, 7.00 pm, 8.00 pm, 9.00 pm, 10.00 pm, midnight, 1.00 am) by chewing on a cotton swab which will be stored in tubes (Salivette™, Sarstedt, Germany). All participants are asked to fill out several questionnaires on the last day of the one-week period (duration = approximately 1 h) and to bring them along at T2. Baseline assessment based on interviews, questionnaires, body parameters/ fitness tests, and neurocognitive tests will be finished at T2 (duration = approximately 4 h for adults and children/parents including breaks) which is scheduled within three weeks after T1 and within one working week after the one-week m-health baseline assessment. Thus, all primary and secondary outcome measures are obtained either at T1, T2, or during the one-week baseline assessment. The randomization takes place at T2. Participants will be instructed in how to use the m-health app for the respective therapy and they will be introduced to all devices. Experimental groups will begin with the interventions the next day, while the control group continues with TAU which lasts for ten weeks. One week after T2, participants will be contacted via email to administer the Rey Auditory Verbal Learning Test (RAVLT) [95] recognition subtest (by sending a link via email to an online survey, 10 min). T3 (mid-intervention assessment) will take place five weeks after T2 (± 3 days). This mid-intervention assessment aims at obtaining the primary and secondary outcome measures during the ongoing trial to get some information on participants dropping out from the study before T4 (duration of T3 = approximately 3 h for adults and children/parents including breaks). Experimental groups will continue with the interventions for another five weeks while the control group continues with TAU. After ten weeks of intervention or TAU (control group) (five weeks after T3 ± 3 days), primary and secondary outcome measures are assessed again (duration = approximately 4 h for adults and children/parents including breaks), followed by a one-week post-intervention assessment with the m-health system including the same parameters as during the baseline m-health assessment. Saliva will be taken during one day of the one-week period (using the same procedure as during the baseline assessment) in the Frankfurt subgroup. One week after T4, participants will be contacted via email to administer the RAVLT recognition subtest. To assess the stability of the therapy effects, the study also includes a follow-up (T5) assessment 12–14 weeks after T4 (T5, duration = approximately 4 h for adults and children/parents including breaks). One week after T5, participants will be contacted via email to administer the RAVLT recognition subtest.

### Sample size calculation

The sample size calculation refers to the primary endpoint, more precisely, the expected change in the clinician-rated observer-blinded IDS-C_30_ total score between T2 and T4 in the intention-to-treat (ITT) population. The sample size calculation is based on the expectation to find a clinically relevant medium effect size (d = 0.5) with a two-sample t-test of at least one of the two interventions compared to TAU on the primary endpoint. As this is a pilot study to establish effect sizes, the sample size is planned at the liberal significance level of α = 0.10 (two-sided) and the power of 1-β = 80%. Taking into consideration the three-group design and an expected drop-out rate of about 30%, n = 219 participants will be allocated and analyzed. Based on the information received from the clinical study centers, it is expected that about 25–35% of the screened participants cannot be included in the study due to violation of inclusion/exclusion criteria. Hence, n = 330 participants will be assessed for eligibility. It can be expected that application of an analysis of covariance in the evaluation will reduce the standard deviation thus increasing the actual power of the study. Sample size calculation was done using ADDPLAN v6.1 [96].

### Recruitment

Recruitment and treatment of participants will be provided at four trial centers, each enrolling 55 participants. King’s College is affiliated with a number of National Health Service foundation trusts across England. The other clinical centers are affiliated or part of University Hospitals and have large outpatient units specialized in ADHD diagnosis and treatment across the life-span, so that the planned recruitment numbers are feasible. Participants are recruited by flyers, public notices, and personal contact from inpatient and outpatient departments of the participating sites, as well as by public announcements, press releases, newspaper advertisements, and Internet/social media campaigns. Measures are in place to mitigate risk in the case of under-recruitment: reimbursement to individual centers will be done in the form of case payment, as stipulated in an agreement before beginning of the trial; pre-financing is carried out on an annual basis. In the case of a participating site falling short of the recruitment plan, the CoCA (EU project Comorbid Conditions of Attention deficit / hyperactivity disorders) steering committee, advised by an independent Data Safety and Monitoring Board (DSMB, see below), reserves the right to stop study participation for this site and either increase recruitment numbers at the remaining sites or include a new recruitment site.

### Assignment of interventions

After written informed consent, participants are successively randomized to one of the three groups (BLT, EI, TAU). At each center, the trial coordinator generates the randomization code using a centralized web-based tool [97] which will be done at T2 after all baseline measurements have been completed in order to ensure allocation concealment. Randomization is a block randomization per center and is done in an allocation ratio of 1:1:1 with fixed block length and stratified for each participating center. The randomization list will be kept in safe and confidential custody at the Institute of Medical Biometry and Informatics (IMBI). Participants withdrawn from the trial will retain their identification codes (e.g. screening number, if already given). New participants must always be allocated a new identification code. As the proposed study is a non-pharmacological therapy intervention study, blinding of participants as well as therapists is not possible. However, the design is observer-blinded. Clinicians, who assess the primary outcome measure, the IDS-C30 total score, and the ADHD Rating Scale total score, are blind to treatment allocation. Therefore, at each site, randomization will be done only by the trial coordinator who is also the only person who will manage the investigator site file (ISF). Clinicians are not involved in the randomization procedure and are not allowed to receive information about group assignment. Clinicians will be provided with data recording sheets and data will be entered into the CRF by the trial coordinator. In addition, participants are instructed not to talk about their intervention at each visit. Adherence to randomization is monitored by an independent clinical on-site monitor, the Coordination Centre for Clinical Trials (KKS), University Hospital Heidelberg.

### Data collection methods

#### Training plans

Group training sessions for all psychologists/psychiatrists involved in the trial from all centers took place at several consortium and kick-off meetings before the trial start. Trainings covered study requirements (e.g. observer-blinded assessment of the primary outcome measure), general information about obtaining research quality data, and recording data (e.g. discussing data collection forms in detail on an item-by-item basis). Data collection forms and the standard operating procedures (SOPs) can be downloaded from the CoCA intranet. The data to be collected and the procedures to be conducted at each visit will be reviewed in detail (see data management and monitoring). Furthermore, intervention manuals were discussed in detail at previous meetings and phone conferences. At subsequent CoCA consortium meetings and during regular monthly phone conferences, clinicians will be monitored with regard to the correct implementation of the manuals and evolving questions can be discussed and solved. The training sessions also included training of the standardized procedures to assess primary and secondary outcome measures. Reliability training of the primary outcome measure will be continued locally following a standard procedure and inter-rater reliability will be assessed during the course of the trial.

#### Participant retention

Once a participant is enrolled or randomized, the study site will make every reasonable effort to follow the participant for the entire study period. Study site staff are responsible for developing and implementing local SOPs to achieve a low rate of loss to follow-up (e.g. reminding participants and parents of the upcoming visits via phone call or email, motivational interviews during visits, etc.).

#### Participant withdrawal

Participants may either withdraw themselves from the intervention, but will stay in the study (I) or the participants may totally withdraw from the trial (II). A third option is that, due to SAEs or other events, the PI decides that the participant has to withdraw from the study.

1. Participants withdrawing at their own request or at request of their legal representative: participants and their legal representatives are allowed to withdraw their consent to participate in the study and the study interventions at any time. The data which were collected before the withdrawal will be used in the statistical analysis.
2. If an individual or the legal representative totally withdraws from the trial and requests the extinction of data, the data cannot be included in the statistical analysis and will be erased from the database.
3. The PI or the local primary investigator needs to decide on the participant’s study withdrawal in the following situations: (i) if, in the principal or primary investigator’s opinion, continuation of the treatment would be detrimental to the participant’s wellbeing; (ii) with admission into a psychiatric hospital; (iii) new occurrence of exclusion criteria under the condition that the security of the participant is thereby endangered. A change in pharmacotherapy is no criterion for withdrawal from the trial. The principal or the local primary investigator decides about withdrawal of participants from the clinical trial or from the investigation in case of occurrence of the criteria mentioned above.

A very low frequency of AEs/SAEs can be expected for BLT and EI. Nevertheless, a DSMB will be installed and safety-relevant events will be reported to this board. Based on the recommendations of the DSMB, the study might be stopped. In all cases, the reason for withdrawal will be recorded in the CRF and in the participant’s medical records. In case of withdrawal of an individual at his/her own request, as far as possible the reason will be asked for as extensively as possible, and documented.

### Data management

The IMBI Heidelberg is responsible for data management comprising all tasks concerning acquisition, processing, and utilization of data with the aim of guaranteeing high quality of the data and providing a valid data basis for the statistical analysis. The system used for data management is validated.

#### Data collection and transmission

Questionnaire, test, and interview data as well as body and fitness parameters will be sent regularly to the IMBI Heidelberg for data entry.

The m-health sensor data will be sent via “FileZilla – The free FTP solution” from all clinical sites to the Karlsruhe Institute of Technology (KIT) and from the KIT to the IMBI Heidelberg by an AES-256 encryption. Therefore, all clinical sites will get their own protected and secure access to save and transfer data. Only the respective clinical site and the KIT will have the authority to enter this access. Afterwards, the data will be transferred to the IMBI Heidelberg in the same protected way. In this case, only the KIT and the IMBI Heidelberg will have the authority to enter this access. Data are stored with pseudonyms only to protect participants. The security of the service is constantly checked by a security scan. The servers are hosted in a secure, ISO 27001 certified environment (datadock Strasbourg).

The app data on the smartphone is also encrypted (256 Bit). If a device gets lost, remote reset is possible. All communication to the web console is highly encrypted with SSL. The data are decrypted as soon as it is in the secure web console. The security of the service is constantly checked by a security scan. The servers are hosted in a secure, ISO 27001 certified environment. Data are stored with pseudonyms only to protect participants. The app data will be transferred from KIT to Heidelberg (IMBI) via “FileZilla” by an AES-256 encryption as well.

Participants are asked to place saliva samples in the freezer or deep-freezer compartment of their fridge and to bring them to the clinic at T2. Saliva samples will be centrifuged, frozen at − 20 °C, stored at the Department of Psychiatry, Psychosomatic Medicine and Psychotherapy, Goethe University, and sent for further analyses and storage to the Department of Psychiatry at University of Rostock. Saliva samples are transferred and stored with pseudonyms only to protect participants.

All findings including clinical data will be documented in the participant’s medical record and in the CRF. The investigator is responsible for ensuring that all sections of the CRF are completed correctly and that entries can be verified against source data (exception: questionnaire data are regarded as source data and part of the CRF at the same time). Any errors should have a single line drawn through them so that the original entry remains legible and the correct data should be entered at the side with the investigator’s signature, date, and reason for change. Self-explanatory corrections need not to be justified. The completed CRF must be reviewed and signed by the investigator named in the trial protocol or by a designated sub-investigator. The original CRF will be transferred to the data management of the IMBI within three weeks after each participant trial visit (T2–T5); one copy will remain with the investigator at the respective clinical sites.

#### Data handling

In order to ensure that the database reproduces the CRFs correctly, the IMBI accomplishes a double entry of data (with the exception of free text) performed by two different persons. The completeness, validity, and plausibility of data are examined by validation programs, which thereby generate queries. The checks to be programmed will be specified beforehand in a data validation plan. The investigator or the designated representatives are obliged to clarify or explain the queries. Any entry and correction in the study database will be reported automatically in an audit file. If no further corrections are to be made in the database, it will be closed (removal of write access) and used for statistical analysis. All data management activities will be done according to the current SOPs of the IMBI.

#### Storage and archiving of data

The database server of the IMBI Heidelberg with the stored data is located in a secure environment and protected by a firewall. During the trial, the data access is restricted to data entry staff and the data manager responsible for the trial. After database closure, the biometrician responsible for the trial gets access to the data for analysis. Backups are performed regularly.

The local investigators will archive all trial data (participant identification code list, source data, and investigator’s file) and relevant correspondence in the ISF. The ISF is kept at each study site. Separate ISFs will be provided for different adolescent and adult departments at the Goethe University, Frankfurt. At all other clinical sites, trial data for adolescents and young adults will be archived in one ISF. The ISF, all source data, and all documents indicated in section 8 of the ICH Consolidated Guideline on good clinical practice (GCP) (as applicable for the present study) will be archived after finalization of the trial according to the local legal regulations, at least for ten years. At the end of the trial, the PI will retain the originals of all CRFs. Trial-related documents will be archived locally. The trial master file will be archived at the Department of Child and Adolescent Psychiatry, Psychosomatics and Psychotherapy, Goethe University Hospital.

### Statistical methods

#### Primary outcome

Statistical methods are used to assess the quality of data, the homogeneity of the treatment groups, the efficacy endpoints, and the safety of the three treatment groups. The confirmatory analysis of the primary endpoint will be conducted on the basis of the ITT population. An additional analysis will be conducted for the per-protocol (PP) population that includes all participants without major protocol violations.

A closed testing procedure will be applied controlling the overall type I error rate at 0.05 (two-sided). A mixed model for repeated measures (MMRM) approach investigating the treatment effect with respect to all three intervention groups will be used. Two-group comparisons I-1 vs TAU, I-2 vs TAU, and I-1 vs I-2 based on contrasts will follow. Baseline IDS-C_30_, age, IQ, sex, treatment, and center will be included as covariates. The MMRM approach models jointly all actual observations without imputing missing data but using the within-participant correlation structure to provide information about unobserved post-baseline primary endpoints. Gender effects are of particular interest. If there are not enough events per category for the different covariates, gender effects will be excluded from the MMRM analysis and investigated in a secondary analysis. The MMRM approach, by which the missing values with respect to post-baseline primary outcomes are dealt with, demonstrates favorable characteristics in terms of type I error rate, power, and bias of estimates compared to alternative methods dealing with missing values, such as last-observation-carried-forward (LOCF) [98–100].

The first (global) hypothesis to be tested states that the change in the IDS-C_30_ total score is equal in all three treatment groups: H0: μI1 = μI2 = μTAU. This hypothesis will be tested at a two-sided level of significance of 5% against the alternative, H1: μI1 ≠ μTAU or μI2 ≠ μTAU or μI1 ≠ μI2. If the first null hypothesis can be rejected, the following three hypotheses for the two group comparisons will be tested simultaneously. One hypothesis to be tested states that the change in IDS-C_30_ total score between baseline (T2) and end of intervention (T4) is equal for I-1 and TAU: H0I1: μI1 = μTAU. This hypothesis will also be tested at a two-sided level of significance of 5% against the alternative hypothesis, H1I1: μI1 ≠ μTAU. A further hypothesis to be tested states that the change in IDS-C_30_ total score between baseline (T2) and end of intervention (T4) is equal for I-2 and TAU: H0I2: μI2 = μTAU. This hypothesis will again be tested at a two-sided level of significance of 5% against the alternative hypothesis, H1I2: μI2 ≠ μTAU. Another hypothesis to be tested states that the change in IDS-C_30_ total score between baseline (T2) and end of intervention (T4) is equal for I-1 and I-2: H0I3: μI1 = μI2. This hypothesis will also be tested at a two-sided level of significance of 5% against the alternative hypothesis, H1I3: μI1 ≠ μI2. The treatment comparisons for these three null hypotheses will be based on the contrasts between I-1 and TAU resp. I-2 and TAU resp. I-1 and I-2 at the end of the intervention (T4).

The above-described confirmatory approach controlling the type I error rate at 0.05 is pursued to enable a proof of efficacy already in this pilot study (for example, if the effect size is higher than anticipated). If the effect size is d = 0.5 as assumed for sample size calculation, the power to reject the null hypothesis of no difference in the primary endpoint comparing one novel intervention to TAU is only 70% (instead of 80%) as planning was performed at the more liberal level of 0.10.

#### Additional analyses

Descriptive methods will be used for the analysis of the secondary outcomes, including the calculation of appropriate summary measures of the empirical distribution (mean, standard deviation, median, minimum and maximum for continuous variables, and frequency in percentages for categorical variables) as well as calculation of descriptive two-sided *p* values. A special focus of the exploratory analysis will be with respect to the time course of the primary as well as the secondary endpoints. Additionally, sensitivity analyses will be conducted for different populations (PP population, appropriate subgroups) and applying different imputation techniques (such as LOCF) for missing values. Further exploratory analyses will be performed to identify intervention effects in subgroups and potential prognostic factors (including hormone levels) for an intervention effect. Furthermore, variables possibly mediating treatment effects such as reward processing, stress reactivity, and compliance with the interventions will be explored. Appropriate regression will be used following the four-step procedure by Baron and Kenny [101]. Graphical methods will be applied to visualize the findings of the study. The safety analysis will be based on the set of all randomized participants who were exposed to study intervention at least once and includes calculation and comparison of frequencies and rates of adverse and serious adverse events reported in the two intervention groups. All analyses will be done using SAS version 9.4 or higher. A statistical analysis plan will be written before the final analysis.

#### Definition of analysis sets

Each participant’s allocation to the different analysis populations (full analysis set according to the ITT principle, PP analysis set, safety analysis set) will be defined and in detail explained in the statistical analysis plan which is finalized before the analysis. During the data review, deviations from the protocol will be assessed as “minor” or “major.” Major deviations from the protocol will lead to the exclusion of a participant from the PP analysis set.

### Monitoring

The study will be monitored by the KKS Heidelberg, a university-based institution which is independent from other trial staff and very experienced in monitoring clinical trials. Monitoring will be done by on-site and off-site visits and frequent communication (letters, telephone, facsimile, email) by a clinical monitor according to SOPs of the KKS and the study-specific monitoring manual. The monitor will ensure that the trial is conducted according to the protocol and regulatory requirements by review of source documents, entries into the CRFs, and essential documents. The monitor will document the visits in a report for the PI as well as the local primary investigator. The site will be provided with a follow-up letter of the findings and the necessary actions to be taken. As the monitoring strategy will consider current aspects of risk-based quality management, frequency of monitoring activities per site will vary depending on recruitment, experience, and general performance, e.g. quality of documentation of the individual trial sites. If there are major findings during monitoring or an audit, the investigational site might be closed by the trial coordinator/PI.

In addition to the standardized monitoring procedures, an independent DSMB has been established that supervises the conduct of the trial and issues recommendations for early termination, modifications, or continuation of the trial, if necessary. The DSMB involves two independent clinical experts and one biometrician for monitoring the progress of the trial and ensure adherence to protocol. The role of the DSMB will be to monitor the progress of the trial and to ensure adherence to protocol. The character of the interventions under investigation makes unexpected SAEs extremely unlikely. For pragmatic reasons, complications recorded at predefined visits, or notified by investigators, will be compiled and reviewed annually. Actions will be taken if, halfway through the trial, clear imbalances regarding the safety of the participants become evident. Also, if evidence from other studies becomes available that definitely favors one or the other intervention and violates therapeutic uncertainty, DSMB members will decide if recruitment needs to be stopped (for further information, see [102]).

### Ethics and dissemination

Before the first individual has been enrolled onto the trial, all ethical and legal requirements were met. Study protocol, participant information, and the respective consent form were approved by the responsible ethical committees before start of the trial. The study protocol was first ethically reviewed and approved by the institutional review board (IRB) of the Medical Faculty, Goethe University, Frankfurt am Main, German (No. 353/16, 13 January 2017). Subsequent approval of this vote was done by the ethical committee of Vall d’Hebron Research Institute, Barcelona, Spain (No. PR(AG)105/2017, 19 April 2017), King’s College London, UK (No. 17/LO/0958, 11 July 2017), and Radboud University Medical Centre, Nijmegen, The Netherlands (No. 2017-3238, 5 October 2017). Any modifications to the protocol which may impact on the conduct of the study, potential benefit of the participant, or may affect participant safety, including changes of study objectives, study design, participant population, sample sizes, study procedures, or significant administrative aspects, will require a formal amendment to the protocol. The IRB of the PI, as well as the IRB of the participating centers and trial registries will be informed of all subsequent protocol amendments which require approval in accordance with local legal requirements.

The procedures set out in this trial protocol, pertaining to the conduct, evaluation, and documentation of this trial, are designed to ensure that all persons involved in the trial abide by GCP (as far as applicable) and the ethical principles described in the current revision of the Declaration of Helsinki. The trial is carried out in keeping with local legal and regulatory requirements, although German Drug Law and Medical Device Law are not applicable. Each site’s PI ensures that all persons assisting with the trial are adequately informed about the protocol, any amendments to the protocol, the trial treatments, their trial-related duties, and functions. The particular local investigator maintains a list of sub-investigators and other appropriately qualified persons to whom he or she has delegated significant trial-related duties. Equally, each local primary investigator ensures that the respective center is responsible for the correct application of the therapy-manuals.

Before being enrolled in the clinical trial, the adolescent’s caretaker(s) and the adolescent participant as well as the young adult participant must consent to the participation of the participant after the nature, scope, and possible consequences of the clinical trial have been explained to them in an understandable oral and written form. Participants as well as their caretakers can withdraw from the study any time without giving a reason. Informed consent will be obtained by the local investigator and stored in the ISF at each study site. A copy of the signed informed consent document will be given to the adolescent’s caretaker(s) and the young adult participant. The documents will be in a language understandable to the individual and his/her caretakers and specify who informed him or her. For this reason, there are three information documents in the presented study: one for participating adolescents; one for the parents/caretakers of the participating adolescents; and one for young adults.

Throughout the trial, participants are pseudonymized. Trial data stored on a computer will be stored in accordance with the local data protection law and will be handled in strictest confidence. Distribution of these data to unauthorized persons is strictly prohibited. The appropriate regulations of local data legislation will be fulfilled in their entirety. Authorized persons (e.g. clinical monitors, auditors) regularly inspect the participant-related data collected during the trial ensuring the data protection law (see monitoring). The local primary investigator of each study site will maintain a personal participant identification list (participant numbers with the corresponding names) to enable records to be identified.

The trial statistician as well as the PI, and, in case of SAEs, members of the DMSB, have access to the final trial set for statistical analyses. After the publication of the primary and secondary outcome measures, all responsible investigators at all study sites will get access to the data to be able to reanalyze the data with regard to specific additional research questions. In case of an external request for replication, the respective statistical analysis will be provided by the trial statistician.

Trial results will be reported to participants, healthcare professionals, the public, patient advocacy groups, and other relevant groups via publications, conferences, press releases, and public talks. Authorship eligibility guidelines according to the International Committee of Medical Journal Editors [103] will be adhered to. No professional writers will be involved in any publication.

## Discussion

We have presented a design and protocol for an RCT of two non-pharmacological interventions—BLT and EI—for the prevention of co-morbid depression and obesity in adolescents and young adults with ADHD. This pilot phase-IIa study will evaluate the feasibility and efficacy of the two manualized ten-week interventions combined with m-health based monitoring and reinforcement in this young patient sample.

The PROUD trial has several strong points. It is the first RCT on the use of two new non-pharmacological interventions implemented to directly target the prevention of depression and obesity—two major co-morbidities of adult ADHD. Existing studies primarily addressed the effects of pharmacological and non-pharmacological treatments on the core ADHD symptoms [14]. Given that co-morbidity is a hallmark of adult ADHD [5], significantly increasing disease burden [12], it is an important and timely goal to develop effective treatments of major co-morbidities as well. Moreover, to date no RCT has evaluated the feasibility and efficacy of non-pharmacological interventions that specifically target the prevention of co-morbid conditions during the potentially sensitive phase of adolescence and young adulthood when adherence to pharmacological treatment is also typically low [27–29].

Physical exercise and BLT are two non-pharmacological interventions which have been well established in the treatment of depression in adults and adolescents [40–42, 68–71]. Recent evidence also suggests their efficacy in the treatment of obesity [47, 48, 73]. Importantly, these treatments were chosen because they are thought to directly modulate two key pathophysiological mechanisms of ADHD—namely a dysregulation of the dopaminergic [37] and circadian systems [57, 58]—that potentially link ADHD to co-morbid depression and obesity [38, 39, 59, 60]. Taking the neurobiological evidence into account, it seems reasonable that these non-pharmacological treatments might be specifically useful in the treatment and prevention of depression and obesity in adolescents and young adults with ADHD. However, systematic interventions studies that target these co-morbid disorders of ADHD are lacking. With regard to BLT, only one open-label trial exists and reported positive effects on core ADHD symptoms and co-morbid depressive symptoms in adult patients with ADHD [75]. With regard to physical exercise, preliminary findings in children with ADHD point to its effectiveness mainly on core clinical ADHD and cognitive symptoms [50]. Furthermore, according to this systematic review [50], higher-quality intervention research is needed including large sample sizes, adequate control groups, observer-blinded assessments, and the use of a wide variety of clinical, cognitive, behavioral, and physical/(neuro)physiological outcome measures. The PROUD study is a prospective RCT that fulfils rigorous methodological requirements by including a large sample of 219 participants at four study centers, a TAU control condition, and observer-blinded assessment of the primary outcome measure. This is a critical aspect because participants cannot be blinded with regard to the intervention. Also, an automated online randomization procedure is clearly defined and reflects the study design with respect to the multicenter study and the group-based randomization. The statistical analysis also takes the different study centers into account. A strong advantage of this trial is the inclusion of several additional secondary outcome measures to assess immediate as well as long-term intervention effects on obesity (measured in terms of several body composition parameters), ADHD-specific symptoms, general psychopathological symptoms, health-related quality of life, neurocognitive functions, chronotype, and body-related measures such as blood pressure and heart rate, physical fitness, and concentrations of hormones. In addition, variables possibly moderating or mediating treatment effects will be explored.

The PROUD trial also bears substantial innovation potential by making use of a mobile technology developed specifically for this trial by the KIT. Both interventions will be supported by the m-health application which has three strong advantages. First, targeting adolescents and young adults with BLT and EI implies specific problems, as this age group usually has little motivation for lifestyle change. The mobile technology is generally viewed very favorably by this age group and therefore it is reasonable that the app-based instruction reminders and reinforcement strategies booster their motivation for change [54]. Second, the sensor allows recording of relevant parameters such as physical activity and light exposure throughout the study phase which makes it possible to monitor compliance during the intervention. Third, the m-health system will be used to assess physical activity, light exposure and additional cognitive-emotional parameters (i.e. reward processing and stress reactivity) online at home pre- and post-intervention. These parameters will then be included in the analyses as variables moderating or mediating the treatment effects.

The manualized interventions, combining BLT and EI with m-health-based monitoring and reinforcement to increase the participant’s motivation and compliance are easy to implement both for the clinicians and the participants. The interventions are portable, cost-effective, and almost entirely free of side effects. Therefore, if the PROUD trial can prove their feasibility and efficacy in adolescents and young adults with ADHD, they have the potential to act as adjuncts to treatment as usual or even as primary treatments in the future.

In conclusion, the high risk for co-morbid depression and obesity for adolescents with ADHD has been increasingly recognized and guidelines for treatment and prevention are urgently needed. The PROUD trial is a rigorous designed RCT on m-health-based BLT and EI to provide first insights into the feasibility and efficacy of these non-pharmacological interventions to prevent co-morbid depression and obesity in adolescents and young adults with ADHD. If at least medium effects can be established with regard to the prevention of depressive symptoms and obesity, a larger scale confirmatory phase-III trial may be warranted.

## Additional files

Additional file 1: SPIRIT Checklist. (DOC 122 kb)

Additional file 2: Definition  and handling of SAEs. SAEs are defined and it is described how SAEs are assessed, reported, and monitored within the PROUD trial. (DOCX 23 kb)

Additional file 3: Secondary outcome measures of the PROUD trial. Detailed description of all secondary outcome measures that will be assessed at baseline, directly after the intervention/continuing TAU (T4), and/or at 12-week follow-up (T5). (DOCX 57 kb)

## Abbreviations

ADHD: Attention-deficit / hyperactivity disorder
AE: Adverse event
EI: Exercise intervention
ASRS: Adult ADHD Self-Report Scale
AUDIT: Alcohol Use Identification Test
BDI-II: Beck Depression Inventory II
BLT: Bright light therapy
BMI: Body mass index
CBT: Cognitive behavioral therapy
CERQ: Cognitive Emotion Regulation Questionnaire
CIRCA: Circadian rhythm
CoCA: Co-morbid conditions of attention-deficit / hyperactivity disorders
CRF: Case report form
DA: Dopamine
DIVA: Diagnostic Interview for ADHD in adults
DSM-5: Diagnostic and Statistical Manual of Mental Disorders, 5th edition
DSMB: Data and Safety Monitoring Board
EHI: Edinburgh Handedness Inventory
EQ-5D-3 L: EuroQol-5 Dimensions-3 Levels
FTND: Fagerström Test for Nicotine Dependence
GCP: Good clinical practice
GHQ-28: General Health Questionnaire
I: Intervention
IDS-C_30_: Inventory of Depressive Symptomatology
IFIS: International Fitness Scale
IMBI: Institute of Medical Biometry and Informatics
IQ: Intelligence quotient
IR: Infrared light
IRB: Institutional Review Board
ISF: Investigator site file
KIT: Karlsruhe Institute of Technology
KKS: Coordination Centre for Clinical Trials (“Koordinierungszentrum Klinische Studien”)
K-SADS-PL: Kiddie-Schedule for Affective Disorders and Schizophrenia -Present and Lifetime Version
LOCF: Last observation carried forward
m-health: Mobile health
MCTQ: Munich Chronotype Questionnaire
MEQ: Morningness–Eveningness Questionnaire
MMRM: Mixed-effects model for repeated measures
NIDA: National Institute on Drug Abuse
PAR-Q: Physical Activity Readiness Questionnaire
PI: Principal investigator
PP: Per protocol
PROUD: Pilot randomized controlled phase-IIa trial on the prevention of comorbid depression and obesity in attention-deficit / hyperactivity disorder
RAVLT: Rey Auditory Verbal Learning Test
RCT: Randomized controlled trial
SAE: Serious adverse event
SCID-I/II: Structured Clinical Interview for psychiatric disorders, based on DSM-IV; part I: psychiatric disorders, part II: personality disorders
SF-36: Short Form Health Questionnaire
SOP: Standard operation procedure
SPIRIT: Standard protocol items: Recommendation for interventional trials
T1 … T5: Time-point 1 …. Time-point 5
TAU: Treatment as usual
UPPS: Impulsive Behaviour Scale
UV: Ultraviolet
VO2max: Maximal oxygen uptake
WAIS: Wechsler Adult Intelligence Scale
WISC: Wechsler Intelligence Scale for Children
WRAADDS: Wender-Reimherr Adult ADHD Symptom Rating Scale
Y(A)SR: Youth (Adult) self-report; YFAS, Yale Food Addiction Scale

## References

[1] Wilens TE, Faraone SV, Biederman J (2004). Attention-deficit/hyperactivity disorder in adults. JAMA..

[2] Simon V, Czobor P, Balint S, Meszaros A, Bitter I (2009). Prevalence and correlates of adult attention-deficit hyperactivity disorder: meta-analysis. Br J Psychiatry..

[3] Polanczyk GV, Willcutt EG, Salum GA, Kieling C, Rohde LA (2014). ADHD prevalence estimates across three decades: an updated systematic review and meta-regression analysis. Int J Epidemiol..

[4] Kooij JJS, Huss M, Asherson P, Akehurst R, Beusterien K, French A (2012). Distinguishing comorbidity and successful management of adult ADHD. J Atten Disord..

[5] Jacob CP, Romanos J, Dempfle A, Heine M, Windemuth-Kieselbach C, Kruse A (2007). Co-morbidity of adult attention-deficit/hyperactivity disorder with focus on personality traits and related disorders in a tertiary referral center. Eur Arch Psychiatry Clin Neurosci..

[6] Meinzer MC, Lewinsohn PM, Pettit JW, Seeley JR, Gau JM, Chronis-Tuscano A (2013). Attention-deficit/hyperactivity disorder in adolescence predicts onset of major depressive disorder through early adulthood. Depress Anxiety..

[7] Yoshimasu K, Barbaresi WJ, Colligan RC, Voigt RG, Killian JM, Weaver AL, et al. Adults with persistent ADHD: gender and psychiatric comorbidities-a population-based longitudinal study. J Atten Disord. 2016; 10.1177/1087054716676342. 10.1177/1087054716676342 PMC5600693 27864428

[8] Cortese S, Moreira-Maia CR, St Fleur D, Morcillo-Penalver C, Rohde LA, Faraone SV (2016). Association between ADHD and obesity: a systematic review and meta-analysis. Am J Psychiatry..

[9] Nigg JT, Johnstone JM, Musser ED, Long HG, Willoughby MT, Shannon J (2016). Attention-deficit/hyperactivity disorder (ADHD) and being overweight/obesity: New data and meta-analysis. Clin Psychol Rev..

[10] Cortese S, Tessari L (2017). Attention-deficit/hyperactivity disorder (ADHD) and obesity: update 2016. Curr Psychiatry Rep..

[11] Nigg JT (2013). Attention-deficit/hyperactivity disorder and adverse health outcomes. Clin Psychol Rev..

[12] Dalsgaard S, Østergaard SD, Leckman JF, Mortensen PB, Pedersen MG (2015). Mortality in children, adolescents, and adults with attention deficit hyperactivity disorder: A nationwide cohort study. Lancet..

[13] Chronis-Tuscano A, Molina BSG, Pelham WE, Applegate B, Dahlke A, Overmyer M (2010). Very early predictors of adolescent depression and suicide attempts in children with attention-deficit/hyperactivity disorder. Arch Gen Psychiatry..

[14] Faraone SV, Biederman J, Spencer TJ, Aleardi M (2006). Comparing the efficacy of medications for ADHD using meta-analysis. Med Gen Med..

[15] Hutchison SL, Ghuman JK, Ghuman HS, Karpov I, Schuster JM (2016). Efficacy of atomoxetine in the treatment of attention-deficit hyperactivity disorder in patients with common comorbidities in children, adolescents and adults: a review. Ther Adv Psychopharmacol..

[16] Bolaños CA, Barrot M, Berton O, Wallace-Black D, Nestler EJ (2003). Methylphenidate treatment during pre- and periadolescence alters behavioral responses to emotional stimuli at adulthood. Biol Psychiatry..

[17] Carlezon WA, Mague SD, Andersen SL (2003). Enduring behavioral effects of early exposure to methylphenidate in rats. Biol Psychiatry..

[18] Biederman J, Monuteaux MC, Spencer T, Wilens TE, Faraone SV (2009). Do stimulants protect against psychiatric disorders in youth with ADHD? A 10-year follow-up study. Pediatrics..

[19] Lee M-J, Yang K-C, Shyu Y-C, Yuan S-S, Yang C-J, Lee S-Y (2016). Attention-deficit hyperactivity disorder, its treatment with medication and the probability of developing a depressive disorder: A nationwide population-based study in Taiwan. J Affect Disord..

[20] Staikova E, Marks DJ, Miller CJ, Newcorn JH, Halperin JM (2010). Childhood stimulant treatment and teen depression: is there a relationship?. J Child Adolesc Psychopharmacol..

[21] Daviss WB (2008). A review of co-morbid depression in pediatric ADHD: etiology, phenomenology, and treatment. J Child Adolesc Psychopharmacol..

[22] Chang Z, D’Onofrio BM, Quinn PD, Lichtenstein P, Larsson H (2016). Medication for attention-deficit/hyperactivity disorder and risk for depression: a nationwide longitudinal cohort study. Biol Psychiatry..

[23] Chen Q, Sjolander A, Runeson B, D’Onofrio BM, Lichtenstein P, Larsson H (2014). Drug treatment for attention-deficit/hyperactivity disorder and suicidal behaviour: register based study. BMJ..

[24] Bangs ME, Emslie GJ, Spencer TJ, Ramsey JL, Carlson C, Bartky EJ (2007). Efficacy and safety of atomoxetine in adolescents with attention-deficit/hyperactivity disorder and major depression. J Child Adolesc Psychopharmacol..

[25] Punja S, Shamseer L, Hartling L, Urichuk L, Vandermeer B, Nikles J (2016). Amphetamines for attention deficit hyperactivity disorder (ADHD) in children and adolescents. Cochrane Database Syst Rev..

[26] Faraone SV, Biederman J, Morley CP, Spencer TJ (2008). Effect of stimulants on height and weight. J Am Acad Child Adolesc Psychiatry..

[27] Frank E, Ozon C, Nair V, Othee K (2015). Examining why patients with attention-deficit/hyperactivity disorder lack adherence to medication over the long term: a review and analysis. J Clin Psychiatry..

[28] McCarthy S, Asherson P, Coghill D, Hollis C, Murray M, Potts L (2009). Attention-deficit hyperactivity disorder: treatment discontinuation in adolescents and young adults. Br J Psychiatry..

[29] Molina BSG, Hinshaw SP, Swanson JM, Arnold LE, Vitiello B, Jensen PS (2009). The MTA at 8 years: prospective follow-up of children treated for combined-type ADHD in a multisite study. J Am Acad Child Adolesc Psychiatry..

[30] Chan E, Fogler JM, Hammerness PG (2016). Treatment of attention-deficit/hyperactivity disorder in adolescents: a systematic review. JAMA..

[31] Vidal R, Castells J, Richarte V, Palomar G, García M, Nicolau R (2015). Group therapy for adolescents with attention-deficit/hyperactivity disorder: a randomized controlled trial. J Am Acad Child Adolesc Psychiatry..

[32] Boyer BE, Geurts HM, Prins PJM, van der Oord S (2015). Two novel CBTs for adolescents with ADHD: the value of planning skills. Eur Child Adolesc Psychiatry..

[33] Sprich SE, Safren SA, Finkelstein D, Remmert JE, Hammerness P (2016). A randomized controlled trial of cognitive behavioral therapy for ADHD in medication-treated adolescents. J Child Psychol Psychiatry..

[34] Young Z, Moghaddam N, Tickle A. The efficacy of cognitive behavioral therapy for adults with ADHD: a systematic review and meta-analysis of randomized controlled trials. J Atten Disord. 2016; 10.1177/1087054716664413. 10.1177/1087054716664413 27554190

[35] Jensen CM, Amdisen BL, Jorgensen KJ, Arnfred SMH (2016). Cognitive behavioural therapy for ADHD in adults: systematic review and meta-analyses. Atten Defic Hyperact Disord..

[36] Antshel KM, Faraone SV, Gordon M (2014). Cognitive behavioral treatment outcomes in adolescent ADHD. J Atten Disord..

[37] Del Campo N, Chamberlain SR, Sahakian BJ, Robbins TW (2011). The roles of dopamine and noradrenaline in the pathophysiology and treatment of attention-deficit/hyperactivity disorder. Biol Psychiatry..

[38] Whitton AE, Treadway MT, Pizzagalli DA (2015). Reward processing dysfunction in major depression, bipolar disorder and schizophrenia. Curr Opin Psychiatry..

[39] van de Giessen E, Celik F, Schweitzer DH, van den Brink W, Booij J (2014). Dopamine D2/3 receptor availability and amphetamine-induced dopamine release in obesity. J Psychopharmacol..

[40] Josefsson T, Lindwall M, Archer T (2014). Physical exercise intervention in depressive disorders: meta-analysis and systematic review. Scand J Med Sci Sports..

[41] Cooney GM, Dwan K, Greig CA, Lawlor DA, Rimer J, Waugh FR (2013). Exercise for depression. Cochrane Database Syst Rev..

[42] Carter T, Morres ID, Meade O, Callaghan P (2016). The effect of exercise on depressive symptoms in adolescents: a systematic review and meta-analysis. J Am Acad Child Adolesc Psychiatry..

[43] Ortega FB, Ruiz JR, Labayen I, Lavie CJ, Blair SN (2018). The Fat but Fit paradox: what we know and don’t know about it. Br J Sports Med..

[44] Ortega FB, Lavie CJ, Blair SN (2016). Obesity and cardiovascular disease. Circ Res..

[45] Waters E, de Silva-Sanigorski A, Hall BJ, Brown T, Campbell KJ, Gao Y (2011). Interventions for preventing obesity in children. Cochrane Database Syst Rev..

[46] Guerra PH, da Silveira JAC, Salvador EP (2016). Physical activity and nutrition education at the school environment aimed at preventing childhood obesity: evidence from systematic reviews. J Pediatr..

[47] Ruotsalainen H, Kyngas H, Tammelin T, Kaariainen M (2015). Systematic review of physical activity and exercise interventions on body mass indices, subsequent physical activity and psychological symptoms in overweight and obese adolescents. J Adv Nurs..

[48] Shaw K, Gennat H, O’Rourke P, Del Mar C (2006). Exercise for overweight or obesity. Cochrane Database Syst Rev..

[49] Cadenas-Sanchez C, Vanhelst J, Ruiz JR, Castillo-Gualda R, Libuda L, Labayen I (2017). Fitness and fatness in relation with attention capacity in European adolescents: The HELENA study. J Sci Med Sport..

[50] Halperin JM, Berwid OG, O’Neill S (2014). Healthy body, healthy mind?: the effectiveness of physical activity to treat ADHD in children. Child Adolesc Psychiatr Clin N Am..

[51] Rommel A-S, Halperin JM, Mill J, Asherson P, Kuntsi J (2013). Protection from genetic diathesis in attention-deficit/hyperactivity disorder: possible complementary roles of exercise. J Am Acad Child Adolesc Psychiatry..

[52] Wigal SB, Nemet D, Swanson JM, Regino R, Trampush J, Ziegler MG (2003). Catecholamine response to exercise in children with attention deficit hyperactivity disorder. Pediatr Res..

[53] Peyrin L, Pequignot JM, Lacour JR, Fourcade J (1987). Relationships between catecholamine or 3-methoxy 4-hydroxy phenylglycol changes and the mental performance under submaximal exercise in man. Psychopharmacology..

[54] Schoenfelder E, Moreno M, Wilner M, Whitlock KB, Mendoza JA (2017). Piloting a mobile health intervention to increase physical activity for adolescents with ADHD. Prev Med Rep..

[55] Kamp CF, Sperlich B, Holmberg H-C (2014). Exercise reduces the symptoms of attention-deficit/hyperactivity disorder and improves social behaviour, motor skills, strength and neuropsychological parameters. Acta Paediatr..

[56] Sánchez-López M, Pardo-Guijarro MJ, Del Campo DG-D, Silva P, Martínez-Andrés M, Gulías-González R (2015). Physical activity intervention (Movi-Kids) on improving academic achievement and adiposity in preschoolers with or without attention deficit hyperactivity disorder: study protocol for a randomized controlled trial. Trials..

[57] Baird AL, Coogan AN, Siddiqui A, Donev RM, Thome J (2012). Adult attention-deficit hyperactivity disorder is associated with alterations in circadian rhythms at the behavioural, endocrine and molecular levels. Mol Psychiatry..

[58] Coogan AN, Baird AL, Popa-Wagner A, Thome J (2016). Circadian rhythms and attention deficit hyperactivity disorder: The what, the when and the why. Prog Neuro-Psychopharmacol Biol Psychiatry..

[59] Wynchank DS, Bijlenga D, Lamers F, Bron TI, Winthorst WH, Vogel SW (2016). ADHD, circadian rhythms and seasonality. J Psychiatr Res..

[60] Vogel SWN, Bijlenga D, Tanke M, Bron TI, van der Heijden KB, Swaab H (2015). Circadian rhythm disruption as a link between Attention-Deficit/Hyperactivity Disorder and obesity?. J Psychosom Res..

[61] McClung CA (2007). Circadian genes, rhythms and the biology of mood disorders. Pharmacol Ther..

[62] Delezie J, Challet E (2011). Interactions between metabolism and circadian clocks: reciprocal disturbances. Ann N Y Acad Sci..

[63] Verwey M, Dhir S, Amir S. Circadian influences on dopamine circuits of the brain: regulation of striatal rhythms of clock gene expression and implications for psychopathology and disease. F1000Res. 2016;5 10.12688/f1000research.9180.1. 10.12688/f1000research.9180.1 PMC5007753 27635233

[64] van der Heijden KB, Smits MG, van Someren EJW, Ridderinkhof KR, Gunning WB (2007). Effect of melatonin on sleep, behavior, and cognition in ADHD and chronic sleep-onset insomnia. J Am Acad Child Adolesc Psychiatry..

[65] van Veen MM, Kooij JJS, Boonstra AM, Gordijn MCM, van Someren EJW (2010). Delayed circadian rhythm in adults with attention-deficit/hyperactivity disorder and chronic sleep-onset insomnia. Biol Psychiatry..

[66] Tamarkin L, Reppert SM, Klein DC (1979). Regulation of pineal melatonin in the Syrian hamster. Endocrinology..

[67] van Maanen A, Meijer AM, van der Heijden KB, Oort FJ (2016). The effects of light therapy on sleep problems: A systematic review and meta-analysis. Sleep Med Rev..

[68] Golden RN, Gaynes BN, Ekstrom RD, Hamer RM, Jacobsen FM, Suppes T (2005). The efficacy of light therapy in the treatment of mood disorders: a review and meta-analysis of the evidence. Am J Psychiatry..

[69] Martensson B, Pettersson A, Berglund L, Ekselius L (2015). Bright white light therapy in depression: A critical review of the evidence. J Affect Disord..

[70] Swedo SE, Allen AJ, Glod CA, Clark CH, Teicher MH, Richter D (1997). A controlled trial of light therapy for the treatment of pediatric seasonal affective disorder. J Am Acad Child Adolesc Psychiatry..

[71] Gest S, Holtmann M, Bogen S, Schulz C, Pniewski B, Legenbauer T (2016). Chronotherapeutic treatments for depression in youth. Eur Child Adolesc Psychiatry..

[72] Nussbaumer B, Kaminski-Hartenthaler A, Forneris CA, Morgan LC, Sonis JH, Gaynes BN (2015). Light therapy for preventing seasonal affective disorder. Cochrane Database Syst Rev..

[73] Beauchamp MT, Lundgren JD. A systematic review of bright light therapy for eating disorders. Prim Care Companion CNS Disord. 2016;18(5). 10.4088/PCC.16r02008 27835724

[74] Fargason RE, Fobian AD, Hablitz LM, Paul JR, White BA, Cropsey KL (2017). Correcting delayed circadian phase with bright light therapy predicts improvement in ADHD symptoms: A pilot study. J Psychiatr Res..

[75] Rybak YE, McNeely HE, Mackenzie BE, Jain UR, Levitan RD (2006). An open trial of light therapy in adult attention-deficit/hyperactivity disorder. J Clin Psychiatry..

[76] Liu F, Kong X, Cao J, Chen S, Li C, Huang J (2015). Mobile phone intervention and weight loss among overweight and obese adults: a meta-analysis of randomized controlled trials. Am J Epidemiol..

[77] Trull TJ, Ebner-Priemer U (2013). Ambulatory assessment. Annu Rev Clin Psychol..

[78] Chan A-W, Tetzlaff JM, Gøtzsche PC, Altman DG, Mann H, Berlin JA (2013). SPIRIT 2013 explanation and elaboration: guidance for protocols of clinical trials. BMJ..

[79] Horne JA, Ostberg O (1976). A self-assessment questionnaire to determine morningness-eveningness in human circadian rhythms. Int J Chronobiol..

[80] http://www.health.gov/paguidelines/. Accessed 14 Oct 2014.

[81] Terman M, Terman JS (2005). Light therapy for seasonal and nonseasonal depression: efficacy, protocol, safety, and side effects. CNS Spectr..

[82] Rush AJ, Gullion CM, Basco MR, Jarrett RB, Trivedi MH (1996). The Inventory of Depressive Symptomatology (IDS): psychometric properties. Psychol Med..

[83] Drieling T, Scharer LO, Langosch JM (2007). The Inventory of Depressive Symptomatology: German translation and psychometric validation. Int J Methods Psychiatr Res..

[84] Helmreich I, Wagner S, Mergl R, Allgaier A-K, Hautzinger M, Henkel V (2011). The Inventory Of Depressive Symptomatology (IDS-C(28)) is more sensitive to changes in depressive symptomatology than the Hamilton Depression Rating Scale (HAMD(17)) in patients with mild major, minor or subsyndromal depression. Eur Arch Psychiatry Clin Neurosci..

[85] Kaufman J, Birmaher B, Brent D, Rao U, Flynn C, Moreci P (1997). Schedule for Affective Disorders and Schizophrenia for School-Age Children-Present and Lifetime Version (K-SADS-PL): initial reliability and validity data. J Am Acad Child Adolesc Psychiatry..

[86] Kooij JJS, Francken MH. Diagnostic Interview for ADHD in adults (DIVA). 2010. http://www.divacenter.eu/DIVA.aspx?id=505. Accessed 8 Aug 2017.

[87] Wittchen HU, Zaudig M, Fydrich T (1997). SKID Strukturiertes Klinisches Interview für DSM-IV Achse I und II Handanweisung.

[88] Kessler RC, Adler L, Ames M, Demler O, Faraone S, Hiripi E (2005). The World Health Organization Adult ADHD Self-Report Scale (ASRS): a short screening scale for use in the general population. Psychol Med..

[89] Marchant BK, Reimherr FW, Robison D, Robison RJ, Wender PH (2013). Psychometric properties of the Wender-Reimherr Adult Attention Deficit Disorder Scale. Psychol Assess..

[90] Bohn MJ, Babor TF, Kranzler HR (1995). The Alcohol Use Disorders Identification Test (AUDIT): validation of a screening instrument for use in medical settings. J Stud Alcohol..

[91] http://www.drugabuse.gov/sites/default/files/pdf/nmassist.pdf. Accessed 16 Aug 2007.

[92] Thomas S, Reading J, Shephard RJ (1992). Revision of the Physical Activity Readiness Questionnaire (PAR-Q). Can J Sport Sci..

[93] Wechsler D (2008). Wechsler Adult Intelligence Scale.

[94] Wechsler D (2003). Wechsler intelligence scale for children – Fourth edition (WISC-IV): The Psychological Corporation.

[95] Schmidt M (1996). The Rey auditory verbal learning test.

[96] http://www.iconplc.com/innovation/addplan/. Accessed 7 Jun 2016.

[97] The Randomizer. http://www.randomizer.at. Accessed 8 Aug 2017.

[98] Mallinckrodt CH, Lane PW, Schnell D, Peng Y, Mancuso JP (2008). Recommendations for the primary analysis of continuous endpoints in longitudinal clinical trials. Drug Inf J..

[99] Lane P (2008). Handling drop-out in longitudinal clinical trials: a comparison of the LOCF and MMRM approaches. Pharm Stat..

[100] Siddiqui O, Hung HMJ, O’Neill R (2009). MMRM vs. LOCF: a comprehensive comparison based on simulation study and 25 NDA datasets. J Biopharm Stat..

[101] Baron RM, Kenny DA (1986). The moderator-mediator variable distinction in social psychological research: conceptual, strategic, and statistical considerations. J Pers Soc Psychol..

[102] CoCA consortium. http://coca-project.eu/about/organisation/. Accessed 14 Aug 2017.

[103] ICMJE. http://www.icmje.org/recommendations/browse/roles-and-responsibilities/defining-the-role-of-authors-and-contributors.html. Accessed 14 Aug 2017.

