# Supplementary material for: Bright light therapy versus physical exercise to prevent co-morbid depression and obesity in adolescents and young adults with attention-deficit / hyperactivity disorder: study protocol for a randomized controlled trial
Source: Trials. 2018 Feb 26;19:140. doi: 10.1186/s13063-017-2426-1 (PMC5828138; doi:10.1186/s13063-017-2426-1)
Supplement: Supplementary file 2 — Definition and handling of SAEs. SAEs are defined and it is described how SAEs are assessed, reported, and monitored within the PROUD trial. (DOCX 23 kb) [file 13063_2017_2426_MOESM2_ESM.docx]

**Additional file 2: Definition** **and handling of (Serious) Adverse Events**

Safety parameters are all (Serious) Adverse Events [(S)AEs] reported by the included participants, or detected by the local investigator during the trial (i.e., occurring after the subject has signed the informed consent document). According to GCP, an AE is defined as follows: Any untoward medical occurrence in a subject participating in a clinical trial. An AE can therefore be any unfavourable and unintended sign (including an abnormal laboratory finding), symptom, or disease, whether or not related to the trial intervention. An AE may be new symptoms/medical conditions, new diagnosis, inter-current diseases and accidents, worsening of medical conditions/diseases existing before clinical trial start, or increase of frequency or intensity of episodical diseases. Surgical procedures themselves are not AEs; they are therapeutic measures for conditions that require surgery. The condition for which the surgery is required may be an AE. Planned surgical measures permitted by the clinical trial protocol and the condition(s) leading to these measures are not AEs, if the condition leading to the measure was present prior to inclusion into the trial. AEs fall into the categories "non-serious" and "serious". A SAE is one that results in death, is life-threatening, requires subject hospitalization or prolongation of existing hospitalization, results in persistent or significant disability/incapacity, is a congenital anomaly/birth defect, or has to be regarded as SAE in the opinion of the investigator. All AEs reported by the subject or detected by the local primary investigator will be collected during the trial and must be documented on the appropriate pages of the CRF. AEs must also be documented in the subject’s medical records. In this trial, all AEs that occur after the subject has signed the informed consent document will be documented on the pages provided in the CRF. All participants who have AEs, whether considered associated with the use of the trial intervention or not, must be monitored to determine the outcome. The clinical course of the AE will be followed up to the time of resolve or normalization of changed laboratory parameters or until it has changed to a stable condition. The intensity of an AE should be assessed by the local primary investigator as mild (i.e, temporary event which is tolerated well by the subject), moderate (i.e., an event which results in discomfort for the subject and impairs his/her normal activity), or severe (i.e., an event which results in substantial impairment of normal activities of subject). For each AE, the local primary investigator will evaluate if coherency with the trial treatment possibly exists (i.e., certain, probable, possible, unlikely, unrelated, or not assessed).

SAEs must be reported by facsimile and by e-mail to the Principal Investigator within 24 hours after the SAE becomes known using the "Serious Adverse Event" form. The initial report must be as complete as possible including details of the current illness and SAE, and an assessment of the causal relationship between the event and the trial treatment needs to be done. The investigator must also inform the site monitor in all cases. The investigator is responsible for notification of SAEs to the responsible institutional review board. During and following a subject’s participation in the trial, the investigator ensures that adequate medical and psychosocial care is provided to a subject for any AEs including clinically significant laboratory values.
