# Supplementary material for: Bright light therapy versus physical exercise to prevent co-morbid depression and obesity in adolescents and young adults with attention-deficit / hyperactivity disorder: study protocol for a randomized controlled trial
Source: Trials. 2018 Feb 26;19:140. doi: 10.1186/s13063-017-2426-1 (PMC5828138; doi:10.1186/s13063-017-2426-1)
Supplement: Supplementary file 3 — Secondary outcome measures of the PROUD trial. Detailed description of all secondary outcome measures that will be assessed at baseline, directly after the intervention/continuing TAU (T4), and/or at 12-week follow-up (T5). (DOCX 57 kb) [file 13063_2017_2426_MOESM3_ESM.docx]

**Additional file 3: Secondary outcome measures of the PROUD trial**

The following secondary outcome measures will be assessed at baseline, directly after the intervention/ continuing TAU (T4) and/ or at 12-weeks follow-up (T5):

1. Change in depressive symptoms
   1. Change in the IDS-C_30_ total score (observer-blinded assessment) is assessed between T2 and T5.
   2. Change in the Beck Depression Inventory (BDI-II) total score between T2 and T4 and between T2 and T5. The BDI-II is widely used self-report scale for persons age 13 and older to assess the severity of depressive symptom [1]. It contains 21 items that are rated on a Likert (0-3 coded) scale.
2. Change in aspects of quality of life
   1. Change in the General Health Questionnaire (GHQ-28) total score between T2 and T4 and between T2 and T5. The GHQ-28 is a self-report to assess the psychological aspect of quality of life which can be administered in individuals aged 14 and older [2]. It incorporates 28 items on four subscales (somatic symptoms, anxiety and insomnia, social dysfunction, and severe depression).
   2. Change in self-reported health status total score as assessed by the EuroQol-5 Dimensions-3 Levels (EQ-5D-3L) between T2 and T4 and between T2 and T5. This is a standardized self-report measure of health status based on five dimensions (each having 3 levels) for adults and children (aged 12 years and older) developed by the EuroQol Research Foundation [3]. Participants also rate their health status on a visual analogue scale in order to quantify the measure of health outcome.
   3. Change in the Short Form Health Questionnaire (SF-36) physical and mental subscores (self-report; 1 week recall version) between T2 and T4. The SF-36 is a well-validated generic measure of health-related quality of life that has been used in studies of many medical and psychiatric conditions in individuals aged 14 and older [4]. It includes 8 subscales and two broad outcomes, a physical and a mental composite.
3. Change in ADHD symptoms and other psychopathology between T2 and T4 and between T2 and T5
   1. Change in ADHD symptom severity total score as assessed by the ADHD Rating Scale. Separate versions exist for children/ adolescents [5] and adults [6]. Both scales are exactly comparable 18-item scales assessing ADHD symptoms (adjusted to DSM-5 criteria) by a 4-point Likert-type severity scale. The scales give a total ADHD score, as well as separate scores for inattentive, hyperactive, and impulsive subscales. The clinician-rated symptom severity for each item will be based on his or her interview with the adult participant based on the Diagnostic Interview for ADHD in adults (DIVA) [7]. For adolescents, symptom severity will be rated for each item based on the Kiddie-Schedule for Affective Disorders and Schizophrenia -Present and Lifetime Version (K-SADS-PL) [8]. To ensure high valid symptom ratings, the K-SADS-PL will be administered by trained clinicians interviewing the adolescent and at least one primary caregiver separately, and finally achieving summary ratings which include both sources of information. Ratings will be done observer-blinded.
   2. Change in self-reported emotional and behavioural problems as assessed by the Youth Self-Report (YSR) and the Adult Self-Report (ASR) (all Y(A)SR subscales T-scores). The Y(A)SR is a self-report scale for adolescents aged 11-18 years old [9] and adults aged 18-59 years old [10] to assess emotional and behavioural problems. The instrument allows an age-adjusted subjective measurement of psychopathology resulting in 10 scales comprising internalizing and externalizing behaviours.
4. Change in circadian rhythm total score between T2 and T4 and between T2 and T5 as assessed by the Munich Chronotype Questionnaire (MCTQ). This is a self-rated scale (including 13 items) to assess sleep behaviour and circadian rhythm [11]. This questionnaire has been developed in ages 6 to > 65 years and categorizes each participant into one of seven chronotype groups.
5. Change in neurocognitive functions between T2 and T4 and between T2 and T5
   1. Change in the Cognitive Emotion Regulation Questionnaire (CERQ) total score. The CERQ is a self-report questionnaire for adolescents and adults aged 12 years and older consisting of 36 items to measures nine different cognitive coping strategies someone uses after having experienced negative events or situations [12]. This questionnaire is chosen to better understand the cognitive emotion regulation strategies associated with depressive symptoms.
   2. Change in short-term and working memory as assessed by two subtests of the Wechsler Adult Intelligence Scale (WAIS-IV) [13]/ Wechsler Intelligence Scale for Children (WISC-IV) [14]: digit span forward and digit span backward. The WISC-IV and WAIS-IV are parallel IQ tests designed to measure intelligence and cognitive ability in children/adolescents (age 6-16, WISC-IV) and adolescents/adults aged 17 and older (WAIS-IV). Changes in digit spans and total scores are assessed.
   3. Changes in the free word recall total scores (immediate and delayed recall) and recognition memory total score (administered a week later via sending a link by email to an online survey) of the Rey Auditory Verbal Learning Test (RAVLT) [15]. The RAVLT is a neuropsychological assessment designed to evaluate verbal memory in participants 6 years of age and older.
6. Change in physical fitness between T2 and T4 and between T2 and T5
   1. Change in physical fitness parameters as assessed by physical fitness tests. General muscular fitness is assessed by measuring maximum handgrip strength with a hand dynamometer with adjustable grip (TKK 5401 Grip D; Takey, Tokyo, Japan) and by measuring lower limb explosive strength with the standing long jump test. The Chester Step Test, a sub-maximal stepping test of aerobic fitness, is used to estimate VO2max. People with a wide range of absolute aerobic fitness levels can be assessed by having adjustable step heights. The validity of these fitness tests have been demonstrated in adolescents and young adults [16–19].
   2. Change in self-reported physical fitness assessed by the International Fitness Scale total scores (IFIS). This is a short self-administered scale composed of five Likert-scale questions to assess physical fitness on different dimensions in adolescents aged 12-17 years old and adults [16].
7. Change in body composition parameters between T2 and T4 and between T2 and T5
   1. Change in Body Mass Index (BMI), waist circumference, waist-to-hip ratio, and body fat percentage calculated based on skinfold thickness measurements using a skinfold caliper (Holtain Skinfold Caliper).
   2. Resting heart rate, systolic, and diastolic blood pressure are measured by an automated digital device (Omron M300 HEM 7121). The device has been validated, and is recommended by the German Hypertension Society [20].
8. Change in parameters measured with the m-Health app between 1-week baseline and 1-week post-intervention assessment: number of steps, movement acceleration, raw scores on Likert scales assessing positive / negative affect, raw scores on visual analog scales assessing event-related reward experience, stress reactivity and inattention; sleep time, and context as assessed by multiple choice questionnaires.
9. Changes in concentrations of melatonin, cortisol, leptin, and ghrelin between T2 and T4 (only assessed in the Frankfurt subgroup).
10. Assessment of possible mediating variables of therapeutic effect at T2 and T4:
    1. Compliance with intervention as measured with the m-Health app ongoing during the intervention.
    2. Reward processing and stress reactivity measured with the m-Health app during 1-week baseline and 1-week post-intervention assessments.
11. Assessment of possible moderating variables of therapeutic effect at baseline (T1)
    1. Chronotype as assessed by the Morningness-Eveningness-Questionnaire (MEQ) [21] at T1. This questionnaire, which was originally designed for adults, consists of 19 questions. The MEQ total score will be used to estimate the individual chronotype and the optimal time point for light exposure.
    2. Light exposure (duration), physical activity (acceleration, number of steps), and mood regulation (reward experience, stress reactivity, positive and negative affect) assessed in real time during a 1-week baseline assessment using the m-Health app combined with light and movement sensors.
    3. Food addiction as assessed by the Yale Food Addiction Scale (YFAS). This self-report questionnaire identifies people with distinctive symptoms indicative of addiction to certain foods. The scale which has been validated for children and young adults [22, 23] contains 25 items in dichotomous and Likert-type format (0-4) that are summed to give a total symptom score which reflects the number of addiction-like criteria endorsed.
    4. Impulsive behavior as assessed by the Impulsive Behavior Scale (UPPS). The UPPS is a 45-item self-report (rated on a 4-point Likert scale) that assesses four subscales (urgency, premeditation, perseverance, sensation seeking) to measure distinct dimensions of impulse behavior [24]. A mean value will be calculated separately for each scale.
    5. Nicotine dependence as assessed by Fagerström Test for Nicotine Dependence (FTND). The FTND is a standard instrument for assessing the intensity of physical addiction to nicotine [25]. The test was designed to provide an ordinal measure of nicotine dependence related to cigarette smoking. It contains six items that evaluate the quantity of cigarette consumption, the compulsion to use, and dependence.
    6. Inclusion of age and gender as possible confounding factors and medication as moderating factor in secondary analyses.

References

1. Beck, AT, Steer RA, Brown GK. Beck Depression Inventory–II (BDI–II). San Antonio, TX: Harcourt Assessment Inc; 1996.

2. Goldberg DP, Hillier VF. A scaled version of the General Health Questionnaire. Psychol. Med. 1979;9:139.

3. EuroQol--a new facility for the measurement of health-related quality of life. Health Policy. 1990;16:199–208.

4. Ware JE, Sherbourne CD. The MOS 36-item short-form health survey (SF-36). I. Conceptual framework and item selection. Med Care. 1992;30:473–83.

5. Dupaul GJ. ADHD rating scale-IV: Checklists, norms, and clinical interpretation. New York: Guilford Press; 1998.

6. Barkley RA. Barkley Adult ADHD Rating Scale-IV (BAARS-IV). New York [etc.]: The Guilford Press; 2011.

7. Kooij, J. J.S. & Francken, M. H. Diagnostic Interview for ADHD in adults (DIVA). 2010. http://www.divacenter.eu/DIVA.aspx?id=505. Accessed 8 Aug 2017.

8. Kaufman J, Birmaher B, Brent D, Rao U, Flynn C, Moreci P, et al. Schedule for Affective Disorders and Schizophrenia for School-Age Children-Present and Lifetime Version (K-SADS-PL): initial reliability and validity data. J Am Acad Child Adolesc Psychiatry. 1997;36:980–8.

9. Achenbach, T. M., Rescorla, L. A. Manual for the ASEBA School-Age Forms & Profiles. Burlington, VT: University of Vermont, Research Center for Children, Youth, & Families; 2001.

10. Achenbach, T. M., Rescorla, L. A. Manual for the ASEBA Adult Forms & Profiles. Burlington, VT: University of Vermont, Research Center for Children, Youth, & Families; 2003.

11. Roenneberg T, Wirz-Justice A, Merrow M. Life between clocks: daily temporal patterns of human chronotypes. J Biol Rhythms. 2003;18:80–90.

12. Garnefski N, Kraaij V, Spinhoven Ph. Negative life events, cognitive emotion regulation and emotional problems. Personality and Individual Differences. 2001:1311–27.

13. Wechsler D. Wechsler Adult Intelligence Scale. 4th ed. San Antonio, TX: Psychological Corporation; 2008.

14. Wechsler D. Wechsler intelligence scale for children – Fourth edition (WISC-IV): The Psychological Corporation. San Antonio, TX: The Psychological Corporation.; 2003.

15. Schmidt M. The Rey auditory verbal learning test. Los Angeles: Western Psychological Services.; 1996.

16. Ortega FB, Ruiz JR, Espana-Romero V, Vicente-Rodriguez G, Martinez-Gomez D, Manios Y, et al. The International Fitness Scale (IFIS): usefulness of self-reported fitness in youth. Int J Epidemiol. 2011;40:701–11.

17. Ortega FB, Silventoinen K, Tynelius P, Rasmussen F. Muscular strength in male adolescents and premature death: cohort study of one million participants. BMJ. 2012;345:e7279.

18. Bennett H, Parfitt G, Davison K, Eston R. Validity of Submaximal Step Tests to Estimate Maximal Oxygen Uptake in Healthy Adults. Sports Med. 2016;46:737–50.

19. Castro-Pinero J, Padilla-Moledo C, Ortega FB, Moliner-Urdiales D, Keating X, Ruiz JR. Cardiorespiratory fitness and fatness are associated with health complaints and health risk behaviors in youth. J Phys Act Health. 2012;9:642–9.

20. German Hypertension Society. https://www.hochdruckliga.de/messgeraete-mit-pruefsiegel.html. Accessed 8 Aug 2017.

21. Horne JA, Ostberg O. A self-assessment questionnaire to determine morningness-eveningness in human circadian rhythms. Int J Chronobiol. 1976;4:97–110.

22. Gearhardt AN, Corbin WR, Brownell KD. Preliminary validation of the Yale Food Addiction Scale. Appetite. 2009;52:430–6.

23. Gearhardt AN, Roberto CA, Seamans MJ, Corbin WR, Brownell KD. Preliminary validation of the Yale Food Addiction Scale for children. Eat Behav. 2013;14:508–12.

24. Whiteside SP, Lynam DR. The Five Factor Model and impulsivity: Using a structural model of personality to understand impulsivity. Personality and Individual Differences. 2001;30:669–89.

25. Heatherton TF, Kozlowski LT, Frecker RC, Fagerstrom KO. The Fagerstrom Test for Nicotine Dependence: a revision of the Fagerstrom Tolerance Questionnaire. Br J Addict. 1991;86:1119–27.
